# Supplementary material for: Feasibility of implementation of simplified management of young infants with possible serious bacterial infection when referral is not feasible in tribal areas of Pune district, Maharashtra, India
Source: PLoS One. 2020 Aug 24;15(8):e0236355. doi: 10.1371/journal.pone.0236355 (PMC7446882; doi:10.1371/journal.pone.0236355)
Supplement: S1 Table — (DOCX) [file pone.0236355.s001.docx]

**Table 1: Differences between the recommendations of management of possible serious bacterial infection (PSBI) in 0-59 days old young infants in the Government of India and WHO guidelines**

|  | **Government of India Guideline 2014 [1]** | **WHO guidelines 2015 [2]** | **Rationale for accepting WHO guideline** |
| --- | --- | --- | --- |
| 1 | Clinical signs of PSBI: | Clinical signs of PSBI: | **In the multi-country Young Infant Study [3,4,5,6]**  Nasal flaring did not increase sensitivity (beyond the 7 signs included)  Grunting did not increase sensitivity (beyond the 7 signs included)  This sign was extremely rare in young infants – thus removed.  Skin infections were not found to be associated with serious outcome. Therefore moved to yellow classification and thus treatment with oral antibiotic. |
|  | Nasal Flaring | Not included |  |
|  | Grunting | Not included |  |
|  | Blood in stools | Not included |  |
|  | 10 or more skin pustules or a big boil | Not included |  |
|  | Temperature >37.5 C | Temperature 38.0 C | The lower temperature cut-off reduces the specificity of the algorithm to identify PSBI. In AFRINEST and SATT studies^3,4,5,6^ temperature cut-off of 38C was used. Those with temperature between 37.5 and 38 C were not treated, and these infants did not have a poor outcome. |
| 2 | When referral is not feasible for PSBI, the guideline recommends same treatment for all these infants:  **Fast breathing in 7-59 days^*^** treated with oral amoxicillin twice daily PLUS IM Gentamicin once daily x 7 days  **Clinical severe infection***¶* treated with oral amoxicillin twice daily PLUS IM Gentamicin once daily x 7 days  **Critical Illness^†^** treated with oral amoxicillin twice daily PLUS IM Gentamicin once daily x 7 days | When referral was not feasible, infants with PSBI **re-classified** into three categories – fast breathing^*^, clinical severe infection^¶^ and critical illness^†^  **Fast breathing in 7-59 days^*^** treated with oral amoxicillin twice daily x 7 days (no gentamicin injection)  **Clinical severe infection and fast breathing in 0-6 days***¶* treated with oral amoxicillin twice daily PLUS IM Gentamicin once daily x 7 days  **Critical Illness^†^** : referral re-enforced and only when referral absolutely refused, treated with IM ampicillin twice daily PLUS Inj Gentamicin once daily x 7 days (no oral antibiotic) | AFRINEST compared IM procaine penicillin plus IM gentamicin with oral amoxicillin alone for young infants with fast breathing alone, and found them to be equivalent.  AFRINEST found that IM gentamicin for 7 days or for 2 days with oral amoxicillin were equivalent. Both options are included in WHO guideline. Many countries have chosen the 2 day option for operational ease and for reaching high coverage.  Oral medicine is not safe to be given to children with critical illness (not able to feed at all, convulsing, unconscious etc.) |
| 3 | Oral amoxicillin recommended in 30-50 mg/kg/day divided into two doses | Oral amoxicillin recommended in high dose: 100 mg/kg/day divided into two doses | AFRINEST and SATT studies used high dose of oral amoxicillin was used to achieve bactericidal levels for most organisms for sepsis in young infants. We have no data on efficacy of lower dose of amoxicillin in treatment of severe newborn infections. |
| 4 | Injection Gentamicin given at dosage of 5 mg/kg body weight once a day | Injection Gentamicin given at dosage of 5 mg/kg body weight once a day |  |

* *Fast breathing -* *Respiratory rate 60 per minute or more*

*† Critical Illness - convulsions, unable to feed at all, no movement on stimulation, unable to cry, bulging fontanelle and cyanosis*

*¶ Clinical severe infection - not feeding well, fever (temperature ≥ 38 °C), low body temperature (< 35.5 °C), severe chest in-drawing, movement only when stimulated*

*We have added in the manuscript in the section on methods that the GOI guideline was brought in line with WHO guideline*

1. Government of India. Operational Guidelines for use of Gentamicin by ANMs for management of sepsis in young infants under specific situations. Ministry of Health and Family Welfare, Government of India, Feb 2014. Available at http://tripuranrhm.gov.in/Guidlines/2606201401.pdf accessed 26th May 2020.
2. Department of Maternal, Newborn, Child and Adolescent Health (MCA), WHO. MANAGEMENT OF THE SICK YOUNG INFANT AGE UP TO 2 MONTHS. WHO Geneva 2015
3. African Neonatal Sepsis Trial (AFRINEST) group, Tshefu A, Lokangaka A, et al. Simplified antibiotic regimens compared with injectable procaine benzylpenicillin plus gentamicin for treatment of neonates and young infants with clinical signs of possible serious bacterial infection when referral is not possible: a randomised, open-label, equivalence trial. Lancet 2015; 385: 1767–76
4. Arican Neonatal Sepsis Trial (AFRINEST) group, Tshefu A, Lokangaka A, et al. Oral amoxicillin compared with injectable procaine benzylpenicillin plus gentamicin for treatment of neonates and young infants with fast breathing when referral is not possible: a randomised, open-label, equivalence trial. Lancet 2015; 385: 1758–66
5. Mir F, Nisar I, Tikmani SS, Baloch B, Shakoor S, Jehan F et al. Simplified antibiotic regimens for treatment of clinical severe infection in the outpatient setting when referral is not possible for young infants in Pakistan (Simplified Antibiotic Therapy Trial [SATT]): a randomised, open-label, equivalence trial. Lancet Glob Health 2017; 5: e177–85

6. Baqui A, Saha S, Ahmed A, Shahidulla M, Quasem I, Roth DE et al. Safety and efficacy of alternative antibiotic regimens compared with 7 day injectable procaine benzylpenicillin and gentamicin for outpatient treatment of neonates and young infants with clinical signs of severe infection when referral is not possible: a randomised open label, equivalence trial. Lancet Glob Heal. 2015; 3: e279–87
