# Supplementary material for: Feasibility of implementation of simplified management of young infants with possible serious bacterial infection when referral is not feasible in tribal areas of Pune district, Maharashtra, India
Source: PLoS One. 2020 Aug 24;15(8):e0236355. doi: 10.1371/journal.pone.0236355 (PMC7446882; doi:10.1371/journal.pone.0236355)
Supplement: S2 Table — (DOCX) [file pone.0236355.s002.docx]

**Supplementary Table 2: Infrastructure of Primary Health Care Centres (PHC) and linked subcenters in the study area**

| **Block** | **Name of PHCs** | **No. of Sub Centres** | **MO** | **ANM** | **MPW** | **ASHA** | **AWW** | **Population** | **Distance to SDH Manchar in kilometres** | **Average distance to referral hospital in kilometres** |
| --- | --- | --- | --- | --- | --- | --- | --- | --- | --- | --- |
| Ambegaon | Adiware | 6 | 2 | 6 | 4 | 23 | 37 | 9066 | 60 | 80 - 140 |
|  | Taleghar | 5 | 2 | 7 | 5 | 22 | 28 | 9771 | 50 |  |
|  | Dimbhe | 7 | 2 | 8 | 7 | 35 | 60 | 23500 | 25 |  |
|  | Total | 18 | 6 | 21 | 16 | 80 | 125 | 42337 | NA |  |
| Junnar | Aptale | 10 | 2 | 13 | 9 | 47 | 66 | 30862 | 55 |  |
|  | Inglun | 7 | 2 | 7 | 7 | 35 | 37 | 16048 | 65 |  |
|  | Madh | 12 | 2 | 12 | 10 | 43 | 57 | 25807 | 63 |  |
|  | Total | 29 | 6 | 32 | 26 | 125 | 160 | 72717 | NA |  |
| Khed | Dehane | 7 | 2 | 7 | 7 | 34 | 37 | 14235 | 65 |  |
|  | Kude | 8 | 2 | 8 | 5 | 26 | 24 | 10653 | 60 |  |
|  | Total | 15 | 4 | 15 | 12 | 60 | 61 | 24888 | NA |  |
| Grand Total | | 62 | 16 | 68 | 54 | 265 | 346 | 139942 | NA |  |

MO- Medical officer, ANM- Auxillary Nurse Midwife, MPW- Multipurpose worker, ASHA - accredited social health activist,

AWW- Anganwadi worker SDH – Subdistrict Hospital
