# Supplementary material for: Feasibility of implementation of simplified management of young infants with possible serious bacterial infection when referral is not feasible in tribal areas of Pune district, Maharashtra, India
Source: PLoS One. 2020 Aug 24;15(8):e0236355. doi: 10.1371/journal.pone.0236355 (PMC7446882; doi:10.1371/journal.pone.0236355)
Supplement: S3 Table — (DOCX) [file pone.0236355.s003.docx]

**Table 3: Birth surveillance and postnatal visits (July 2017 to March 2019) in Pune**

| **Birth surveillance** | **Total number** | |
| --- | --- | --- |
| Population of catchment area | 139942 | |
| Total live births recorded | 3071 | |
| Total live births followed in the study - N (%) | 2001 (65) | |
| **Post-natal visits by day by ASHAs, N (% of live births)** | |  |
|  | N (%) out of total live births | N (%) out of those followed |
| No of postnatal visits on Day 1 | 1777(57.9) | 1777(88.8) |
| No of postnatal visits on Day 3 | Not available | Not available |
| No of postnatal visits on Day 7 | 1316(42.9) | 1316(65.7) |
| No of postnatal visits on Day 14 | 1429(46.2) | 1420(70.9) |
| No of postnatal visits on Day 21 | 1400(45.6) | 1400(69.9) |
| No of postnatal visits on Day 28 | 1382(45.0) | 1382(69.0) |
| No of postnatal visits on Day 42 | Not available | 1380(68.9) |
| No of postnatal visits on Day 59 | 1360(44.9) | 1360(67.9) |
