# Supplementary material for: Feasibility of implementation of simplified management of young infants with possible serious bacterial infection when referral is not feasible in tribal areas of Pune district, Maharashtra, India
Source: PLoS One. 2020 Aug 24;15(8):e0236355. doi: 10.1371/journal.pone.0236355 (PMC7446882; doi:10.1371/journal.pone.0236355)
Supplement: S4 Table — (DOCX) [file pone.0236355.s004.docx]

**Table 4: Baseline survey of knowledge of Accredited Social Health Activists (ASHA) and counselling received by mothers on newborn care and danger signs**

| **KNOWLEDGE OF ASHAS**  **(N = 61, EXCEPT WHERE INDICATED OTHERWISE)** | | **KNOWLEDGE AND EXPERIENCE OF MOTHERS**  **(N = 121)** | |
| --- | --- | --- | --- |
| **Indicator** | **n (%)** | **Indicator** | **n (%)** |
| Knowledge about breastfeeding initiation within 1 hour of delivery (N = 61) | 58 (96.7) | Newborn care counselling received | 109 (90) |
| Knowledge about exclusive breastfeeding for first 6 months | 60 (98.4) | **Counseling received about danger signs** | |
| Knowledge about overall care of newborns | 52 (85.2) | a.    Not able to feed since birth/stopped feeding well or not feeding at | 72 (59.5) |
| **Knowledge about specific signs** | | b.  Convulsions/ Seizures | 64 (52.9) |
| Infant not able to feed since birth/stopped feeding well or not feeding at all | 34 (55.7) | c.    Lower Chest Movements (Severe Chest Indrawing) | 53 (43.8) |
| Convulsions/ Seizures | 29 (47.5) | d.  Hot to touch | 88 (72.7) |
| Lower Chest Movements (Severe Chest Indrawing) | 28 (45.9) | e.  Feels cold to touch | 64 (52.9) |
| Hot to touch | 41 (67.2) | f.   Movement only when stimulated | 45 (37.2) |
| Feels cold to touch | 32 (52.5) | g.    Fast Breathing (breaths 60/minute of more | 54 (44.6) |
| Movement only when stimulated | 18 (29.5) |  |  |
| Fast Breathing (breaths 60/minute or more) | 22 (36.1) |  |  |
