# Supplementary material for: Feasibility of implementation of simplified management of young infants with possible serious bacterial infection when referral is not feasible in tribal areas of Pune district, Maharashtra, India
Source: PLoS One. 2020 Aug 24;15(8):e0236355. doi: 10.1371/journal.pone.0236355 (PMC7446882; doi:10.1371/journal.pone.0236355)
Supplement: S2 Questionnaire — (PDF) [file pone.0236355.s006.pdf]

Implementation Research on management of Possible Serious Bacterial Infection in young infants where referral is not feasible through strengthening of existing Home Based newborn care program of the Government of India

BASELINE SURVEY
MOTHER QUESTIONNAIRE

Section I: Identification भाग-1: पहचान

1.1	Site ID: Haryana1/Himachal Pradesh2/Maharashtra3/Uttar Pradesh4<br>साइट क्रमॉकः हरियाणा1/ हिमाचल प्रदेश2/महाराष्ट्र3/उत्तर प्रदेश4 [ ]		
1.2	District Name:<br>जनपद का नामः	1.3	Block Name:<br>ब्लाक का नामः
1.4	CHC Name:<br>सामुदायिक स्वास्थ्य केन्द्र नाम	1.5	PHC Name:<br>प्राथमिक स्वास्थ्य केन्द्र नामः
1.6	Sub Centre Name:<br>उप केन्द्र का नामः	1.7	Village Name:<br>गाँव का नामः
1.8	ASHA Name:<br>आशा का नामः	1.9	ASHA Mobile Number:<br>आशा का मोबाइल नं0: [ ][ ][ ][ ][ ][ ][ ][ ][ ][ ]
1.10	MCTS ID of Mother : Available1/Not Available Today2 माँ का एमसीटीएस क्रमाँक उपलब्ध1/आज अनुपलब्ध2 [ ]		
1.11	MCTS ID of Mother:<br>माँ का एमसीटीएस क्रमाँकः [ ][ ][ ][ ][ ][ ][ ][ ][ ][ ][ ][ ][ ][ ][ ][ ][ ][ ]		
1.12	MCTS ID of Child बच्चें का एमसीटीएस क्रमाँक :<br>Available1 /Not Available Today2 उपलब्ध1/आज अनुपलब्ध2 [ ]		
1.13	MCTS ID of Child : बच्चें का एमसीटीएस क्रमाँक [ ][ ][ ][ ][ ][ ][ ][ ][ ][ ][ ][ ][ ][ ][ ][ ][ ][ ]		

Section 2: Socio Demographic Characteristics भाग-2: सामाजिक जनसांख्यिकीय विवरण

**Bio-Social Profile of Family परिवार का जैव सामाजिक प्रारूप**			
2.1	Religion धर्म	Hindu1/ Muslim2 /Other3 (specify)___________<br>हिन्दू1/मुस्लिम2/अन्य3 (उल्लेख करें)..................	[ ]
2.2	Caste जाति	General1 /OBC2 /SC3/ST4<br>सामान्य1/अन्य पिछड़ा वर्ग2/अनुसूचित जाति3/ अनुसूचित जनजाति4	[ ]
2.3	Type of family<br>परिवार का प्रकार	Nuclear1/Joint2<br>एकल1/संयुक्त2	[ ]
2.4	Number of family members परिवार के सदस्यों की संख्या		[ ][ ]
2.5	Total family expenditure per month<br>कुल पारिवारिक मासिक व्यय	(Write '999999' if not known)<br>(अगर नहीं पता तो '999999' लिखें)	[ ][ ][ ][ ][ ][ ]
2.6	Total family monthly saving<br>कुल पारिवारिक मासिक बचत	• write '000000' in case of no monthly saving<br>अगर कोई मासिक बचत नहीं तो '000000' लिखे<br>• write '999999' if unknown<br>अगर पता नहीं तो '999999' लिखें	[ ][ ][ ][ ][ ][ ]
2.7	Total loan on family on date of interview<br>कुल पारिवारिक ऋण साक्षात्कार के दिनांक तक	• write '000000' in case of no loan<br>लिखें '000000' अगर कोई कर्ज नही<br>• Write '999999' if unknown<br>अगर नहीं पता तो '999999' लिखें	[ ][ ][ ][ ][ ][ ]
**Characteristics of Parents माता-पिता का विवरण**			
2.8	Age of Mother<br>माता की उम्र	(completed years) (पूर्ण वर्षो में)<br>Check Mother Child Protection (MCP) Card) (एम०सी0पी0 कार्ड के अनुसार)	[ ][ ]
2.9	Education of Mother (completed)<br>माता की पूर्ण शिक्षा	Illiterate1/Primary level2/Middle school3/ Highschool4/Intermediate5/Graduate6/Postgraduate7/Professional8<br>अशिक्षित1/प्राथमिक2/माध्यमिक3/हाईस्कूल4/इण्टरमीडिएट5/स्नातक6/परास्नातक7/व्यवसायिक8	[ ]
2.10	Occupation of Mother<br>माता का व्यवसाय	Housewife1/Un-skilled worker2/Skilled worker3/Farmer4/Shop owner5/Clerical6/Professional7<br>गृहणी1/अकुशलकारीगर2/कुशल कारीगर3/किसान4/दुकानदार5/लिपिक6/व्यवसायिक7	[ ]

2.11	Age of Father<br>पिता की उम्र	(completed years) (पूर्ण वर्षो में)[ ] [ ]
2.12	Education of Father (completed)<br>पिता की पूर्ण शिक्षा	Illiterate$_1$/Primary level$_2$/Middle school$_3$ /High school$_4$/Intermediate$_5$/Graduate$_6$/Postgraduate$_7$/Professionals$_8$<br>अशिक्षित$_1$/प्राथमिक$_2$/माध्यमिक$_3$/हाईस्कूल$_4$/इण्टरमीडिएट$_5$/स्नातक$_6$/परास्नातक$_7$/व्यवसायिक$_8$ [ ]
2.13	Occupation of Father<br>पिता का व्यवसाय	Unemployed$_1$/ Un-skilled worker$_2$/Skilled worker$_3$/Farmer$_4$/Shop owner$_5$/Clerical$_6$/Professional$_7$<br>बेरोजगार$_1$/अकुशलकारीगर$_2$/कुशल कारीगर$_3$/किसान$_4$/दुकानदार$_5$/लिपिक$_6$/व्यवसायिक$_7$ [ ]
**Details of Index Child चिन्हित बच्चें का विवरण**		
2.14	Age आयु	दिन days [ ] [ ]
2.15	Gender लिंग	Male$_1$/Female$_2$ पुरूष$_1$/महिला$_2$ [ ]
2.16	Birth order<br>जन्म का क्रम	[ ] [ ]
2.17	Weight at birth जन्म के समय वनज<br>*(Check Records –MCP Card)*<br>(एम०सी०पी०कार्ड के अनुसार)	*(in grams)* ग्राम में<br>[ ] [ ] [ ] [ ]<br>*(Write 9999 if not known)*<br>(जानकारी प्राप्त न होने पर 9999 लिखें)<br>[ ]<br>*Tick √ here when weight not available in MCPC card and recorded as told by mother* (सही का चिन्ह (√) लगाये यदि वजन एम0सी0पी0सी0 कार्ड में उपलब्ध नहीं है और माता द्वारा मौखिक बताया गया)
2.18	Classify according to weight<br>(for office use only)<br>वजन के आधार पर वर्गीकरण (कार्यालय हेतु)	Assessment [ ]<br>Normal weight$_1$/Low birth weight$_2$/Very low birth weight$_3$/no assessment$_9$<br>सामान्य वजन$_1$/कम वजन$_2$/ बहुत कम वजन$_3$/ बहुत कम वजन$_3$/मूल्यांकन नहीं$_9$
2.19	Gestational age गर्भ की अवधि<br>*(Check Records –MCP Card)* (एम०सी०पी० कार्ड के अनुसार)	a. Date of LMP before pregnancy गर्भधारण से पूर्व मासिक की अंतिम तिथि<br>[ ] [ ]/ [ ] [ ]/[ ][ ][ ][ ]<br>dd mm yyyy<br>दिन माह वर्ष<br>*(Write 99/99/9999 if not date known/not available)*<br>(रिकार्ड उपलब्ध न होने पर 99/99/9999 लिखें)<br><br>b. Date of delivery डिलवरी की तिथि<br>[ ] [ ]/ [ ] [ ]/[ ][ ][ ][ ]<br>dd mm yyyy<br>दिन माह वर्ष<br><br>c. Gestational age *(In Weeks)* [ ] [ ]<br>(for office use only) (कार्यालय हेतु)<br>[Write 99 if gestational age could not be calculated assessment could not be done. 99 लिखे यदि गर्भावधि की उम्र का मूल्यांकन सम्भव नहीं हैं]
2.20	Classify gestational Age<br>गर्भावधि उम्र में वर्गीकृत (कार्यालय हेतु)<br>(for office use only)	Assessment मूल्यांकन [ ]<br>Term$_1$/Preterm$_2$/Post term$_3$/No Assessment$_9$<br>समय से$_1$/समयपूर्व$_2$/समय पश्चात$_3$/मूल्यांकन नही$_9$
2.21	What was mother`s perception about the child at the time of birth?<br>जन्म के समय माँ की बच्चें के बारे में क्या राय थी?	Normal$_1$/Weak$_2$/Very weak$_3$<br>सामान्य$_1$/कमजोर$_2$/अत्यधिक कमाजोर$_3$ [ ]

Section 3: Antenatal Care (ANC) in Index Neonate
भाग-3 प्रसवपूर्व देखभाल (ANC) चिन्हित नवजात के सन्दर्भ में

**Pregnancy registration and ANC गर्भधारण का पंजीकरण और (ANC)**		
3.1	Was there any contact of mother with ASHA during antenatal period?<br>क्या गर्भावस्था के दौरान माँ की आशा से कोई मुलाकात हुयी थी ?	Yes$_1$ हाँ$_1$<br>No$_2$ नही$_2$ [ ]
3.2	Was the ANC registered?<br>क्या इस गर्भावस्था का प्रसव पूर्व देखभाल का पजीकरण हुआ था?	Yes$_1$ हाँ$_1$<br>No$_2$ नही$_2$ (Q.3.6 पर जायें) [ ]
3.3	What was the month of pregnancy at the time of registration?<br>पंजीकरण के समय गर्भावस्था का कौन सा माह था?	[ ] माह

3.4	Where was the ANC registration done?<br>प्रसव पूर्व देखभाल का पंजीकरण कहाँ हुआ था?	At the village₁/ Sub-centre₂/PHC₃/ CHC₄/Bal Mahila Chikatsalaya (BMC)₅/ Other₆Specify: ________<br>गाँव में₁/उपकेन्द्र₂/प्राथमिक स्वास्थ्य केन्द्र₃/सामुदायिक स्वास्थ्य केन्द्र₄/बाल महिला चिकित्सालय₅/ अन्य₆स्पष्ट करे__________	[ ]
3.5	Did mother receive any antenatal care during pregnancy of the index neonate from the place of registration?<br>जिस जगह माँ का पंजीकरण हुआ क्या वहाँ गर्भावस्था के दौरान नवजात के सन्दर्भ में प्रसव पूर्व देखभाल दी गई?	Yes₁ हाँ₁<br>No₂ नही₂	[ ]
3.6	Total no. of antenatal visits anywhere done by the mother मां द्वारा कहीं भी की गई कुल प्रसव पूर्व भ्रमणों की संख्या		[ ][ ]
**Counseling about newborn care and danger signs during antenatal/ postnatal period**<br>**प्रसवपूर्व/प्रसव पश्चात नवजात की देखभाल और खतरों के लक्षणों के सम्बन्ध में परामर्श**			
3.7	Did you get counseling on newborn care during the antenatal visits?<br>क्या आपको नवजात की देखभाल हेतु प्रसव पूर्व परामर्श मिला था?	Yes₁ हाँ₁<br>No₂ नही₂ (go to 3.9)	[ ]
3.8	Who gave this counseling during antenatal period ?<br>आपको प्रसव पूर्व परामर्श किसके द्वारा प्रदान किया गया?	ASHA₁/ANM₂/staff nurse ₃/government doctor₄/ private doctor₅/ Other ₆ Specify....................<br>आशा₁/ए०एन०एम०₂/स्टाफ नर्स₃/सरकारी चिकित्सक₄/ निजी चिकित्सक₅/अन्य कोई₆ स्पष्ट करें..	[ ]<br>[ ]<br>[ ]<br>[ ]<br>[ ]
3.9	Did you get counseling on newborn care during postnatal visits<br>क्या आपको प्रसव पश्चात नवजात की देखभाल के बारे में पराशर्म मिला?	Yes₁ हाँ₁<br>No₂ नही₂ (go to 3.11.1)	[ ]
3.10	Who gave you this counseling during postnatal period ?<br>आपको प्रसव पश्चात परामर्श किसके द्वारा प्रदान किया गया?	ASHA₁/ANM₂/staff nurse ₃/government doctor₄/ private doctor₅/ Other₆ Specify.................................<br>आशा₁/ए०एन०एम०₂/स्टाफ नर्स₃/सरकारी चिकित्सक₄/निजी चिकित्सक₅/अन्य कोई₆/स्पष्ट करें। __________	[ ]<br>[ ]<br>[ ]<br>[ ]<br>[ ]

3.11	**What counseling on the following points young infant care was given to you during the antenatal/postnatal visits**<br>निम्नलिखित बिन्दुओं में से किन-किन बिन्दुओं पर आपको प्रसवपूर्व/प्रसव पश्चात भ्रमण के दौरान शिशु की देखभाल के सम्बन्ध में परामर्श मिला	**3.11.1 ANTENATAL**<br>प्रसव पूर्व<br>मिली देखभाल के आगे सही का निशान√ लगाये अन्यथा क्रास x का निशान	**3.11.2 POSTNATAL**<br>प्रसव पश्चात<br>मिली देखभाल के आगे सही का निशान√ लगाये अन्यथा क्रास x का निशान
	a. Cord Care नाल की देखभाल	[ ]	[ ]
	b. Eye Care आँखों की देखभाल	[ ]	[ ]
	c. Breast Feeding स्तनपान	[ ]	[ ]
	d. Wrapping बच्चें को लपेटना	[ ]	[ ]
	e. Skin to skin contact त्वाचा से त्वाचा का लगाव	[ ]	[ ]
	f. Bathing नहलाना	[ ]	[ ]
	g. Routine Immunization नियमित टीकाकरण	[ ]	[ ]
	**h.** 102/108 Ambulance Service 102/108 एम्बुलेंस सेवा	[ ]	[ ]

3.12	**What information was given on Danger Signs in newborn during the Antenatal/Postnatal Visits?**<br>क्या आपको प्रसवपूर्व/प्रसव पश्चात भ्रमणों के दौरान शिशु में खतरों के लक्षणों से सम्बन्धित निम्नलिखित में से कोई सूचना दी गयी थी?	**3.12.1 ANTENATAL**<br>**प्रसव पूर्व**<br>मिली देखभाल के आगे सही का निशान लगाये अन्यथा क्रास का निशान	**3.12.2 POSTNATAL**<br>**प्रसव पश्चात**<br>मिली देखभाल के आगे सही का निशान लगाये अन्यथा क्रास का निशान
	a. Not able to feed since birth/stopped feeding well or not feeding at all जन्म से स्तनपान करने में असमर्थ/स्तनपान अच्छे से करना बंद कर दिया या विलकुल भी स्तनपान नही कर रहा	[ ]<br>**प्रसव पूर्व**	[ ]<br>**प्रसव पश्चात**
	b. Convulsions/ Seizures ऐठना/झटके	[ ]	[ ]

	c. Lower Chest Movements (Severe Chest Indrawing) पसली धंसना	[ ]	[ ]
	d. Hot to touch छूने पर गर्म महसूस होना	[ ]	[ ]
	e. Feels cold to touch छूने पर ठन्डा महसूस होना	[ ]	[ ]
	f. Movement only when stimulated केवल उदद्दीपन पर हिलना डुलना	[ ]	[ ]
	g. Fast Breathing (breaths 60/minute of more) तेज सांस लेना (सांस 60/प्रति मिनट और अधिक)	[ ]	[ ]
	h. Jaundice पीलिया	[ ]	[ ]
	i. Diarrhea दस्त	[ ]	[ ]
	j. Pustules (10 or more) or one large focus of infection मवाद से भरे दाने (10 और अधिक) अथवा एक बड़ा फोड़ा	[ ]	[ ]
	k. Grunting कराहना	[ ]	[ ]

Section 4: Intra-natal and Post-natal Care भाग-4 प्रसव के दौरान व प्रसव के पश्चात देखभाल

4.1	**Date of delivery** *(Check Records –MCP Card)*<br>प्रसव का दिनाँक (एम०सी०पी० कार्ड को देखे)	[ ][ ]/ [ ][ ]/[ ][ ][ ][ ]<br>dd दिन mm माह yyyy वर्ष
4.2	**Place of delivery:**	Home$_1$ (go to 4.5)<br>Govt health facility$_2$<br>Private health facility$_3$<br>Other$_4$ Speify:__________<br>प्रसव का स्थानः<br>घर पर$_1$ (4.5 पर जाये)<br>सरकारी स्वास्थ्य केन्द्र$_2$<br>निजी स्वास्थ्य केन्द्र$_3$<br>अन्य$_4$ स्पष्ट करें............. [ ]
4.3	**Duration of stay in health facility after delivery** *(in days)*<br>प्रसव के पश्चात स्वास्थ्य केन्द्र पर ठहरने की अवधि दिनो में	[ ][ ] days
4.4	**If, duration of stay in health facility <1 day** *(in hours)(write 99 if not applicable)*<br>यदि प्रसव के पश्चात एक दिन से कम ठहरा हो तो घंटों में बताये घंटे में (लिखें '99' यदि लागू नहीं)	[ ] [ ]hours
4.5	**Mode of delivery:** Normal vaginal delivery$_1$/Assisted$_2$/Caesarian section$_3$<br>प्रसव का प्रकारः सामान्य प्रसव$_1$/सहायता प्राप्त$_2$/शल्य क्रिया द्वारा$_3$	[ ]
	**ASHA Home Visit Related Information** आशा के गृह भ्रमण से संबधित सूचना	
4.6	Did you get information of home visit provision to you or your baby in the postnatal period through ASHA (within 42 days of birth)?<br>क्या आपको यह बताया गया था कि बच्चे के जन्म से 42 दिनों तक अलग-अलग समय पर आशा आपके घर आती रहेगी?	Yes$_1$/हाँ$_1$<br>No$_2$ /नही$_2$<br>(go to 4.8) [ ]
4.7	Person who gave you this information:<br>AWW$_1$/ASHA$_2$/ANM$_3$/Doctor$_4$/Other$_5$ specify……………………<br>किस व्यक्ति के द्वारा आपकों यह सूचना प्राप्त हुयी?<br>आँगनवाडी कार्यकत्री$_1$/आशा$_2$/ए०एन०एम०$_3$/चिकित्सक$_4$/अन्य$_5$ स्पष्ट करे................	[ ]
4.8	How many HBNC visits are scheduled to be completed by ASHA for this index child as on date of interview? (to filled by the interviewer): साक्षात्कार की तिथि तक चिहिन्त शिशु के लिए कितनी एच0बी0एन0सी0 विजिट प्रस्तावित हैं (साक्षात्कारकर्ता द्वारा भरे जाने हेतु)	[ ]
4.9	How many of these HBNC visits have been made by ASHA for this child till the date of interview*(If `No` visit was done write 0)* उत्तरदाता के अनुसार साक्षात्कार की तिथि तक आशा द्वारा कितनी एच0बी0एन0सी0 विजिट पूर्ण कर ली गयी।	[ ]
4.10	Documentation of home visits done by ASHA for the index infant:<br>चिन्हित बच्चों के लिए आशा द्वारा किए गए गृह भ्रमण की तालिका<br>• Tick √ all visit done (किये गए सभी भ्रमण पर सही √ करें)<br>• Cross × all visit not done (यदि सभी भ्रमण न किये गये तो क्रास × कर ें)<br>• Write 'NA' if the visits are not scheduled as per age child age (NA लिखे यदि भ्रमण आयु अनुसार सुनिश्चित नहीं हुआ ें)<br>• HI if infant hospitalized (HI लिखें यदि बच्चा अस्पताल में भर्ती था ें)<br>• HM if mother hospitalized (HM लिखें यदि माता अस्पताल में भर्ती थी ें)	

**Day of ASHA visit** आशा भ्रमण का दिन	**Day 1** दिन 1	**Day3** दिन 3	**Day7** दिन 7	**Day14** दिन 14	**Day 21** दिन 21	**Day 28** दिन 28	**Day 42** दिन 42	**Un-scheduled Visit1** अनिर्धारित भ्रमण1 दिन लिखें	**Un-scheduled Visit2** अनिर्धारित भ्रमण2 दिन लिखें
4.10.1. Post natal Examination of mother									

प्रसव पश्चात माता की जाँच									
4.10.2. Examination of newborn नवजात की जाँच									
4.10.3 Weight of Young Infant बच्चें का वजन									
4.10.4 Temperature of Young Infant बच्चें का तापमान									
4.10.5 Verbal enquiry related to young infant health शिशु के स्वास्थ सम्बन्धित ली गयी मौखिक जानकारी									
4.10.6 Remarks if any: टिप्पणी यदि कोई है									

यदि कोई भी भ्रमण न हुआ हो तो अनुभाग 5 पर जायें।

4.11	Did ASHA wash her hands with soap and water before examining your baby? क्या आपके बच्चें की जाँच के पहले आशा ने अपने हाथों को साबुन और पानी से साफ किया था?	Yes1/No2 हाँ1/नही2 [ ]
4.12	What information did the ASHA give you during the first Home Visit **(Day1 /or Day3)**? प्रथम गृह भ्रमण के दौरान आशा ने आपको क्या जानकारी दी? (दिन 1 या दिन 3 पर)	

**4.12.1 On breast feeding** (Tick all responses given) स्तनपान के बारें में (दिए गये सभी विकल्पों पर टिक √ करें)	• Baby must be only breastfed and no other things should be given such as water, ghutti, honey etc. शिशु को केवल स्तनपान करवायें तथा पानी, घुट्टी, शहद, आदि जैसी कोई और चीजें न दें	[ ]
	• Any other thing given may cause diarrhea किसी अन्य चीज के देने से डायरिया हो सकता है	[ ]
	• Other milks such as cow/goat has less iron that may cause anemia गाय/बकरी जैसे अन्य जानवर के दूध में आयरन कम होता है जिससे अनीमिया हो सकता है	[ ]
	• Do not know/ No information given by ASHA पता नही/आशा के द्वारा कोई भी जानकारी नही दी गयी	[ ]
**4.12.2On infant positioning during breast feeding** (Tick all responses given) स्तनपान के समय शिशु की सही स्थिति के बारे में (दिए गये सभी विकल्पों पर टिक √ करें)	• Close to mother's body नवजात को माता के शरीर के नजदीक रखना	[ ]
	• Facing Mother with nose opposite to nipple बच्चें का मुँह माता की ओर तथा नाक निप्पल की तरफ लगाये रखें	[ ]
	• Hold the infant well supported बच्चें को अच्छें से पकडकर सहारा देना	[ ]
	• Infant head and body in straight line बच्चें का सिर और शरीर एक सीधी रेखा में रहे	[ ]
	• Do not know/ No information given by ASHA पता नही/आशा द्वारा कोई जानाकारी नही दी गई	[ ]
**4.11.3 On infant-breast attachment** (Tick all responses given) माँ और बच्चें के स्तनपान के जुडाव के बारें में (दिए गये सभी विकल्पों पर टिक √ करें)	• Mother should touch her infant`s lip with her nipple मां को बच्चें के होठ को अपने निप्पल से स्पर्श करवाना चाहिए	[ ]
	• Mother should wait until infant's mouth is open मां बच्चें के मुंह खुलने का इंतजार करे।	[ ]
	• Aim the infant's lower lip below the nipple ध्यान रखें नवजात का निचला होठ निप्पल के नीचे रहे।	[ ]
	• Do not know/ No information given by ASHA पता नही/आशा द्वारा कोई जानाकारी नही दी गई।	[ ]
**4.11.4 On Frequency of breast feeding** (Tick one option given) स्तनपान कितनी-कितनी देर पर कराना चाहिए (दिए गये सभी विकल्पों पर टिक √ करें)	• Every 2 hour प्रति 2 घंटे में	[ ]
	• On demand बच्चें की माँग पर	[ ]
	• Less than 8 times/day प्रतिदिन 8 बार से कम	[ ]
	• More than or equal to 8 times/day प्रतिदिन 8 बार या उससे ज्यादा	[ ]
	• Do not know/ No information given by ASHA पता नही/आशा द्वारा कोई जानाकारी नही दी गई।	[ ]

**4.11.5 Timing of first bath** (Tick all responses given) प्रथम स्नान का समय (दिए गये सभी विकल्पों पर टिक √ करें)	• Two days after birth जन्द के दो दिन बाद<br>• 7 days after birth जन्म के 7 दिन<br>• Any other specify ……………………. अन्य कोई स्पष्ट करें………………….<br>• Do not know/ No information given by ASHA पता नही/आशा द्वारा कोई जानाकारी नही दी गई।	[ ]<br>[ ]<br>[ ]<br>[ ]
**4.11.6 On wrapping of baby** (Tick all responses given) बच्चें को लपेटने का तरीका (दिए गये सभी विकल्पों पर टिक √ करें)	• The infant be completely covered as per the weather to maintain at optimum temperature बच्चें का सामान्य तापमान बनए रखने के लिए, मौसम के अनुसार उसे पूरी तरह से लपेट कर रखें।<br>• The infant be covered by cap and socks बच्चें को टोपा एवं मोजा पहनाकर रखें।<br>• The infant should be wrapped by warm cloth बच्चें को गर्म कपडें से लपेटकर रखे।<br>• The wrapping and clothes should be changed when wet गीला होने पर कपडे बदलते रहे।<br>• Do not know/ No information given by ASHA पता नही/आशा द्वारा कोई जानाकारी नही दी गई।	[ ]<br>[ ]<br>[ ]<br>[ ]<br>[ ]
**4.11.7 Skin to skin contact** (Tick one option given) त्वचा से त्वचा का स्पर्श (दिए गये सभी विकल्पों पर टिक √ करें)	• The infant should be kept with skin to skin contact to keep baby warm बच्चें का तापमान बनाये रखने के लिए त्वचा से त्वचा का स्पर्श देते रहे।<br>• Do not know/ No information given by ASHA पता नही/आशा द्वारा कोई जानाकारी नही दी गई।	[ ]<br>[ ]
**4.11.8 On cord care** (Tick all responses given) नाल/नाभि की देखभाल (दिए गये सभी विकल्पों पर टिक √ करें)	• Nothing should be applied on cord नाभि के ऊपर कोई चीज नही लगायें।<br>• Care of the cord should be taken till it dries and falls-off नाभि की देखभाल तब तक करें जब तक की वह सूखकर झड़ न जायें।<br>• Do not know/ No information given by ASHA पता नही/आशा द्वारा कोई जानाकारी नही दी गई।	[ ]<br>[ ]<br>[ ]
**4.11.9 On eye care** (Tick all responses given) आंखों की देखभाल के लिए (दिए गये सभी विकल्पों पर टिक √ करें)	• No '*kajal*' or any other home remedies be applied on the eyes आंखों पर काजल या घरेलू चीजें न लगायें।<br>• If any infection or 'pus' discharge is seen then contact to ANM/Asha and seek for adequate medication किसी भी प्रकार के संक्रमण एव पस के रिसाव के देखे जाने पर ए0एन0एम0/आशा से सम्पर्क करें एवं उचित उपचार करवायें।<br>• Do not know/ No information given by ASHA पता नही/आशा द्वारा कोई जानाकारी नही दी गई।	[ ]<br>[ ]<br>[ ]
**4.11.10 Immunization** (Tick all responses given) टीकाकरण (दिए गये सभी विकल्पों पर टिक √ करें)	• Regular immunization details would be provided from the time to time for immunization as per the program of the village गँाव के कार्यक्रम में अनुसार समय-समय पर नियमित टीकाकरण की जानकारी दी।<br>• Immunization would be done by the ANM at a particular place or time टीकाकरण ए0एन0एम0 द्वारा निर्धारित स्थान एवं समय पर किया जायेगा।<br>• Do not know/ No information given by ASHA पता नही/आशा द्वारा कोई जानाकारी नही दी गई।	[ ]<br>[ ]<br>[ ]
**4.11.11 Hand washing** (Tick all responses given) हाथ धोने की विधि(दिए गये सभी विकल्पों पर टिक √ करें)	• The Infant should be touched only after washing the hands with soap by anybody किसी को भी बच्चें को स्पर्श करने से पहले हाथों को साबुन से अच्छे से साफ करना चाहिए।<br>• Avoid touching of the infant by any sick person किसी भी बीमार व्यक्ति को बच्चें का स्पर्श नही करना चाहिए।<br>• Do not know/ No information given by ASHA पता नही/आशा द्वारा कोई जानाकारी नही दी गई।	[ ]<br>[ ]<br>[ ]

**4.11.12 Danger sign in newborn** नवजात शिशु में खतरें के लक्षण<br>(दिए गये सभी विकल्पों पर टिक √ करें)	• Not able to feed since birth/stopped feeding well or not feeding at all जन्म से स्तनपान नहीं कर पा रहा। आज की तारीख में पहले की अपेक्षा में स्तनपान कम कर दिया/बिल्कुल भी स्तनपान न करना<br>• Convulsions/ seizures ऐंठना/आना<br>• Lower Chest Movements (severe chest indrawing) पसली चलना/ सीना धंसना<br>• Hot to touch छूने पर गर्म महसूस होना<br>• Feels cold to touch छूने पर ठंडा महसूस होना<br>• Movement only when stimulated सिर्फ छूने पर बच्चें का हिलना-डुलना<br>• Fast Breathing (breaths 60/minute of more) तेज-तेज सांसें चलना<br>• Jaundice पीलिया<br>• Diarrhea डायरिया/दस्त होना (पतली टट्टी आना)<br>• Pustules (10 or more) or one large focus of infection 10 या ज्यादा पस के दाने या एक बड़ा फोड़ा<br>• Grunting कराहना/संक्रमण<br>• Do not know/ No information given by ASHA पता नही/आशा द्वारा कोई जानाकारी नही दी गई।	[ ]<br>[ ]<br>[ ]<br>[ ]<br>[ ]<br>[ ]<br>[ ]<br>[ ]<br>[ ]<br>[ ]<br>[ ]<br>[ ]
**4.11.13 What to do in case of identification of danger sign in newborn (in verbatim)** शिशु में गम्भीर बीमारी के लक्षण पाने पर क्या करना चाहिए, के लिए आशा द्वारा किया गया विवरण। (शब्दशः लिखें)	 **Translate to English (for office entry)**	

Section 5: Breast Feeding Practices
भाग- 5 स्तनपान कराने का अभ्यास

5.1	**How soon after the birth you breastfed the baby?** जन्म के कितने समय पश्चात आपने शिशु को स्तनपान करया?	Less than 1 hour1 / 1-4 hours2 / 4- 24 hours3/ >24 hours4/ Never5<br>एक घंटे से कम1/1-4 घंटे2/4-24 घंटें3/>24 घंटे4/कभी नही5	[ ]
5.2	**If never breastfed than why?**<br>यदि कभी स्तनपान नही कराया तो क्यों? विवरण लिखें।<br>**Note verbatim**	--<br>--<br>--<br>(English Translation)<br>--<br>--<br>--	
5.3	**Did you give anything other than breast milk immediately after birth?**<br>जन्म के तुरन्त बाद क्या आपने शिशु को माँ के दूध के अतिरिक्त कुछ और दिया?	Not given1/ Honey or `*ghutti*` or jaggery2 / Water3/ Any other4 (specify)______________<br>नही दिया1/शहद या घुट्टी या गुड2/पानी 3/अन्य 4 उल्लेख करे..........	[ ]
5.4	**Did you give the baby the first milk?** क्या आपने शिशु को अपना पहला पीला गाढा दूध पिलाया?	Yes1 हाँ1 **(go to Q. 5.6)**<br>No2 नही2	[ ]
5.5	**If NO, give reasons for it**<br>यदि नही, इसका कारण बतायें।	It is unsafe1/ Due to social customs2/ Advised to discard it3/Others4 (specify) ……………………..<br>यह असुरक्षित है1/सामाजिक रीति-रिवाज के कारण2/इसको फेंकने की सलाह दी गयी3/ अन्य4 उल्लेख करें.........................	[ ]
5.6	**How frequently did you breastfeed your baby?** कितने	On demand 1/Every 2 hours2 / Less than 8 times per	[ ]

	समय के अन्तराल पर आप अपने बच्चें को दूध पिलाती है?	day3/More than or equal to 8 times per day4<br>बच्चें की माग पर1/ प्रति दो घंटें पर2/प्रतिदिन 8 बार से कम3/प्रति दिन 8 बार या उससे अधिक4	
5.7	**Is the baby exclusively breasted fed (not even water)?** क्या आपने बच्चें का सिर्फ स्तनपान ही कराया? (पानी भी नही)	Yes1(**go to Q. 5.9)** यदि हाँ1, (प्रश्न संख्या 5.9 पर जाये)<br>No2 नही2	[ ]
5.8	**If baby is not breastfed, then which method you are using to feed the baby?** यदि बच्चें ने स्तनपान नही किया, तब बच्चे को आहार देने हेतु आप कौन सी विधि का प्रयोग कर रही है।	Spoon feeding1/ Bottle feeding2/Others3 specify ________________________<br>चम्मच द्वारा1/बोतल द्वारा2/अन्य कोई3उल्लेख करें...............	[ ]
5.9	**What is the frequency of urination of baby?** बच्चा कितने-कितने अन्तराल पर पेशाब करता है।	After every feed1/ 1 – 4 times per day2/ 5 – 10 times per day3/Other4 Specify______________<br>प्रत्येक आहार के बाद1/प्रतिदिन 1-4 बार2/प्रतिदिन 5-10 बार3/ अन्य कोई4 उल्लेख करें...............	[ ]

Section 6: Experience of Serious Illness in Young Infants

भाग-6 चिन्हित नवजात शिशु में गंभीर बीमारी की पहचान

6.1	**Did your baby have any serious illness till date after birth?** क्या आपके बच्चें को जन्म से लेकर अभी तक कोई गंभीर बामारी हुई?	Yes1 हाँ1<br>No2 नही2 (end interview) साक्षात्कार समाप्त करे।	[ ]
6.2	If yes, then on what day of life यदि हाँ, जन्म के कितने दिन बाद		Day [ ][ ]
6.3	What was total duration of illness		Days [ ][ ]
6.4	What symptoms were observed ?<br>कौन-कौन से लक्षण दिखायी दिये	....<br>..<br>..<br>..<br>(English Translation)<br>..<br>..<br>..	
6.5	**Was Treatment taken for the illness बीमारी हेतु क्या उपचार लिया गया** Yes1/No2 (end interview)		[ ]
6.6	Write Verbatim for illness scenario and treatment taken. Note when illness started, symptoms of illness, duration of illness, treatment was inpatient/outpatient, details of treatment and outcome at first place of treatment. Then note same things for second and third places of treatment?<br>बीमारी के परिदृश्य और लिये गये इलाज का कथनानुसार उल्लेख करें। विस्तारपूर्वक लिखे कि बीमारी कब शुरू हुई, बीमारी के लक्षण क्या थे, बीमारी की अवधि क्या थी, बाह्य रोगी/भर्ती द्वारा किया क्या इलाज किया गया और इलाज क्या निष्कर्ष निकला। ये सभी बिन्दु प्रथम, द्वितीय और तृतीय स्थान पर दिये गये उपचार के बारे में भी व्यक्त करें।<br>..<br>..<br>..<br>..<br>..<br>..<br>..<br>..<br>..		

	……………………………………………………………………………………………<br>……………………………………………………………………………………………
6.7	**1st Person/Place of treatment प्रथम व्यक्ति/स्थान जहां से उपचार लिया गया है।** [ ]

6.7.1 **Person Consulted*: किस व्यक्ति से सलाह ली गयी? (परामर्शदाता)** [ ]

6.7.2 What did the consulted person do: Gave Treatment1(go to 6.7.4.)/Referred2 (go to 6.7.3) [ ]
सलाह देने वाले व्यक्ति ने क्या किया उपचार किया1(कृपया प्रश्न 6.7.4 पर जाये)/संदर्भित किया2 (कृपया प्रश्न 6.7.3 पर जाये)

6.7.3 Details of Referral	6.7.4 Details of Treatment
(a) Type of referred facility**: संदर्भित केन्द्र का प्रकार [ ]<br>(b) write name of the helath facility also<br>संदर्भित केन्द्र का नाम भी दें<br>……………………………………<br>……………………………………	(a) Place where treatment was given**: उपचार का स्थान [ ]<br>(b) Treatment type: Inpatient1/ Outpatient2/Not applicable3 [ ]<br>(c) Give the details of the treatment: उपचार का पूर्ण विवरण दें।<br>(also write diagnosis as on discharge ticket in case of inpatient admission)<br>……………………………………………………………………<br>……………………………………………………………………<br>(d) **Total duration of treatment at this place:** उपचार की अवधि<br>**days [ ] [ ]**<br>**(e) Outcome of treatment:** उपचार का परिणाम [ ]<br>Completely cured1 *(If cured, go to Q. 6.10)* /Not cured2/ Died3 (go to Q. 6.10)/ Treament continues till date4 (go to Q. 6.10)<br>पूर्ण रूप से स्वस्थ हुआ1(यदि स्वस्थ हुआ तो प्रश्न 6.10 पर जाये)/स्वस्थ नही हुआ2/मृत्यु हो गयी3 (प्रश्न 6.10 पर जाये)/अभी तक उपचार चल रहा है4 (प्रश्न 6.10 पर जायें)

6.8	**2nd Person/Place of treatment दूसरा व्यक्ति/स्थान जहां से उपचार लिया गया है।** [ ]

	6.8.1 **Person Consulted*:** किस व्यक्ति से सलाह ली गयी? (परामर्शदाता)	[ ]
	**6.8.2 What did the consulted person do:** Gave Treatment1(give to 6.8.4. /Referred2 (go to 6.8.3)<br>सलाह देने वाले व्यक्ति ने क्या किया उपचार किया1/संदर्भित किया	[ ]

6.8.3 Details of Referral	6.8.4 Details of Treatment
(a) Type of referred facility**: संदर्भित केन्द्र का प्रकार [ ]<br>(b) write name of the helath facility also संदर्भित केन्द्र का नाम भी दें<br>……………………………………<br>……………………………………	(a) Place where treatment was given**: उपचार का स्थान [ ]<br>(b) Treatment type: Inpatient1/ Outpatient2/Not applicable3 [ ]<br>(e) Give the details of the treatment: उपचार का पूर्ण विवरण दें।<br>(also write diagnosis as on discharge ticket in case of inpatient admission)<br>…………………………………………………………………………<br>…………………………………………………………………………<br>(c) **Total duration of treatment at this place:** उपचार की अवधि **days [ ] [ ]**<br>**(e) Outcome of treatment:** उपचार का परिणाम [ ]<br>Completely cured1 *(If cured, go to Q. 6.10)* /Not cured2/ Died3 (move to Q. 6.10)/ Treament continues till date4 (move to Q. 6.10)<br>पूर्ण रूप से स्वस्थ हुआ1(यदि स्वस्थ हुआ तो प्रश्न 6.10 पर जाये)/स्वस्थ नही हुआ2/मृत्यु हो गयी3 (प्रश्न 6.10 पर जाये)/अभी तक उपचार चल रहा है4 (प्रश्न 6.10 पर जायें)

6.9 3rd Person/Place of treatment तृतीय व्यक्ति/स्थान जहां से उपचार लिया गया है। [ ]

6.9.1 **Person Consulted*: किस व्यक्ति से सलाह ली गयी? (परामर्शदाता)** [ ]

6.9.2 What did the consulted person do: Gave Treatment1(give to 6.9.41/Referred2 (go to 6.9.3) [ ]
सलाह देने वाले व्यक्ति ने क्या किया उपचार किया1/संदर्भित किया2

6.6.3 Details of Referral	6.9.4 Details of Treatment
**Give details of the referred facility**:** संदर्भित केन्द्र का पूर्ण विवरण दें। [ ]<br>……………………………………<br>……………………………………<br>**write name of the helath facility also**<br>संदर्भित केन्द्र का नाम भी दें	(a) Place where treatment was given**: उपचार का स्थान [ ]<br>(b) Treatment type: Inpatient1/ Outpatient2/Not applicable3 [ ]<br>(c) Give the details of the treatment: उपचार का पूर्ण विवरण दें।<br>(also write diagnosis as on discharge ticket in case of inpatient admission)<br>…………………………………………………………………………<br>…………………………………………………………………………<br>(d) Total duration of treatment at this place: [ ] [ ]<br>**(e)** Outcome of treatment**:** उपचार का परिणाम<br>Completely cured1 *(If cured, go to Q. 6.10)* /Not cured2/ Died3 (move to Q. 6.10)/ Treament continues till date4 (move to Q. 6.10)<br>पूर्ण रूप से स्वस्थ हुआ1(यदि स्वस्थ हुआ तो प्रश्न 6.10 पर जाये)/स्वस्थ नही हुआ2/मृत्यु हो गयी3 (प्रश्न 6.10 पर जाये)/अभी तक उपचार चल रहा है4 (प्रश्न 6.10 पर जायें)

6.10	Total Duration of treatment (indays) उपचार की कुल समयावधि नीचे(दिनों)	[ ] [ ] [ ]
6.11	Total approximate out of pocket money spent on treatment INR<br>उपचार में कुल खर्च हुई अनुमानित धनराशि	[ ][ ][ ][ ]
6.12	Did you use transport service to seek care for sick young inftant<br>क्या आपने बीमार शिशु के उपचार हेतु परिवहन सुविधाओं का उपयोग किया?	Yes1 **हाँ1** [ ]<br>No2 (end interview) नही2 साक्षात्कार समाप्त [ ]
6.13	Which transport did you use to seek helath care for sick infant?<br>आपने अपने शिशु की स्वास्थ देखभाल के लिए कौन से वाहन का इस्तेमाल किया?	Ambulance service 1021/ Ambulance service 1082/Personal Vehicle3/ Private vehicle-Village4/Paid Vehicle/Taxi5 [ ]<br>एम्बुलेंस सुविध 1021/एम्बुलेंस सुविधा 1082/व्यक्तिगत वाहन3/ गाँव का व्यक्तिगत वाहन4/ खर्चे पर वाहन/टैक्सी5

Person consulted * सलाह देने वाला व्यक्ति (परामर्शदाता)	Place of treatment **/Referred Facility** चिकित्सा का स्थान/संदर्भित केन्द्र
ASHA1/ANM2/ AWW3/Private Doctor4/Village Based Doctor5/Faith Healer6/ government doctor7/Self8/Any other9 specify …………………<br>आशा1/ए0एन0एम02/आंगनवाडी कार्यकत्री3/निजी चिकित्सक4/गाँव का चिकित्सक5/झाड-फूक करने वाला6/सरकारी चिकित्सक7/स्वंय द्वारा8/अन्य स्पष्ट करें9 ………….	Home1/PHC2/CHC3/District Hospital4/ Medical College5/Private Hospital6/Home & Hospitalboth7<br>घर1/प्राथमिक स्वास्थ्य केन्द्र2/सामुदायिक स्वास्थ्य केन्द्र3/जिला चिकित्सालय4/मेडिकल कालेज5/निजी अस्पताल6/घर एवं अस्पताल दोनो7

Interview Details		
1.	Interviewer Name<br>साक्षात्कारकर्ता का नाम	……………………………………<br>(Name in capital letters) नाम अंग्रेजी के बड़े अक्षरों में
2.	Signature Interviewer<br>साक्षात्कारकर्ता का हस्ताक्षर	
3.	Date of interview<br>साक्षात्कार की तिथि	[ ] [ ]/[ ] [ ]/[ ] [ ]<br>dd mm yyyy<br>दिन माह वर्ष
4.	Supervisor Name पर्यवेक्षक का नाम	……………………………………<br>(Name in capital letters) नाम अंग्रेजी के बड़े अक्षरों में
5.	Signature of Supervisor पर्यवेक्षक का हस्ताक्षर	
6.	Date of validation:	[ ] [ ]/[ ] [ ]/[ ] [ ]<br>dd mm yyyy<br>दिन माह वर्ष
7.	Data Entry Operator:	[ ] [ ](Initial of name only) केवल नाम का प्रथम अक्षर
8.	Signature Data entry operator<br>डाटा एन्ट्री आपरेटर का हस्ताक्षर	
9.	Date of Data Entry<br>डाटा एन्ट्री की तिथि	[ ] [ ]/[ ] [ ]/[ ] [ ][ ]<br>dd mm yyyy<br>दिन माह वर्ष
