# Supplementary material for: Feasibility of implementation of simplified management of young infants with possible serious bacterial infection when referral is not feasible in tribal areas of Pune district, Maharashtra, India
Source: PLoS One. 2020 Aug 24;15(8):e0236355. doi: 10.1371/journal.pone.0236355 (PMC7446882; doi:10.1371/journal.pone.0236355)
Supplement: Attachment — Submitted filename: Kemhrc pune_PSBI_Reviewers Report_DR Abadi Leul apr 01 2020.docx [file pone.0236355.s007.docx]

**Abadi Leul (Reviewer)**

Thank you for the opportunity. This is an interesting manuscript entitled **“**Simplified management of young infants with possible serious bacterial infection (PSBI) when referral is not feasible in tribal areas of Pune district, Maharashtra, India: Implementation research for demonstration of feasibility and scale up**”**

This topic is timely and addresses new born problems mainly PSBI in order to identify and treat most newborns in their community where referral is not feasible. Hence it is a timely research topic. I want to appreciate the researchers. Having said this I will present my review in details.

General comment

Please check typology, spacing and font sizes. Stick to the PLOS guideline for manuscript writing.

Titles of tables also need to be as per the PLOS guideline.

**Abstract**

Abstract is well summarized

Lines 54 - 55: Coverage rate was 87.5%. How did it come? You did not show it in your result section. Needs revisit.

Line 55 – 56: Please keep consistency. You started with percentage for coverage and referral acceptance. Mention also the same for treatment success or death rate. It seems that 2 out of the 10 CSI/CI treated have died. I understand that the main aim of the implementation research is to identify barriers and facilitators not effectiveness (the regimen is known to be effective). Even then try to keep consistency.

The fact that almost 49% of your cases were only fast breathing pneumonia in 7 – 59 days and all were treated successfully needs mention in the abstract.

**Introduction**
Lines 68 – 69: Reference number five says 92 % of all neonatal infections (not illnesses) are caused by bacterial infection. Neonatal infection is responsible for 46% of neonatal illnesses. Check and correct please

Line 83: Here please either mention all the regimens used in the AFRINEST and SATT studies or remove either procaine penicillin or Amoxicillin. Mention also Pneumonia in 7 – 59 days can be managed effectively without the need for referral as you have implemented this approach as well.

Lines 86 – 87: Please check your reference. Reference number 17 and 18 is about home based new born care but could not get the section which describes about PSBI.

**Methods**

Lines 113 – 114: The outcome is not clear. What do you mean when you state “All or at least 80% of identified sick young infants will receive treatment.” This outcome is not clear. It means for me whatever the rate of identification you will provide at least 80% treatment. One of the challenges in management of neonatal infections is most of them are not being identified. It is not problem of provision of treatment if identified. Of course referral acceptance is an obstacle to get treatment. I would suggest to re write the two out comes as **objective**

1. **To identify at least __ % of all expected PSBI cases**
2. **To provide adequate treatment to at least ___% of those identified.**

The same lines better to avoid **all or** at least are enough

Line115: I see no difference in defining adequate treatment as taking full doses of amoxicillin (10 doses) and five days of injectable gentamicin. In case there is a difference elaborate.

**Design:**

Lines 124 – 125: Which WHO guideline is this. Put a reference.

There are also two options that I know in the WHO guideline. It seems that you choose to use the seven days regimen that comprises 7 days gentamicine injections and twice oral amoxicillin per day for 7 days. Provide justification to use this regimen.

## **Policy dialogue for implementing adapted guidelines**

Line148: Could not see supplemental table 1.Is it in a different attachment than the figures.

Line 158 – 159: What is the difference between RST and TSU? Don’t they have the same task? It is not clear why you divided them into two. I feel the RST is in the TSU. If that is the case IT and TSU may be enough to make things clear.

## **Study area**

Better to re write to keep the flow. Also give a summarized picture of the study area. Total population covered. Expected live births in the catchment area in 20 months

Available health facilities from the lowest level to the highest level in the district. How are they staffed under normal circumstances based on the government policy eg. What are the staffs available at the sub centers PHC, rural hospitals and district hospital? What can they do? Distance between each level of care and catchment population for each tier level.

Line 177: check the number.

## **Baseline survey and mapping of referral system**

General the following should have been included in the base line assessment

The existing services in the PHC and sub center? Activities in relation to PSBI cases? Whether they are identifying these cases and what do they do.

You should have also assessed the MOs the same way as ANM/ASHA

Line 185: what are ANMs? Don’t start with abbreviation in the first time.

Line 86 – 87: what are ASHAs? You didn’t describe them before?

**Line 194: IT team activities**

I don’t see the importance of this title. You can continue with the training and orientation you are describing about training of the master trainers from the TSU.

**Line 200:** Reference 21 is the 2019 WHO guideline**.** The training was given in 2017. Please recheck this.

Line 206: Fig. 3 Needs rearrangement. Based on the WHO guideline PSBI includes FB in young infants 7 – 59 days as well. Please check and correct.

**Identification of sick young infants**

Line 217: What is this higher level? You have Rural and district Hospitals. Are these the referral sites or you have a tertiary level. If different one please provides its distance and the neonatal services in this level somewhere.

**Referral to tertiary health care facilities**

Line 225: It is not clear why you were bypassing the rural and district hospitals. Please explain why this happened. What is the purpose of these hospitals if they don’t serve as referral sites for PSBI cases?

Line 226: Was this the pre referral regimen for India. Is it different from the WHO recommendation which was giving Gentamicine and oral Amoxicillin?

**Treatment when referral is not feasible or referral is not recommended**

Lines 234 – 236: Are you sure you gave oral Amoxicillin and gentamicin injection for CI. Is this in your guideline? Please separate CSI from CI. I feel it is by mistake. You may give ampicillin injection instead of amoxicillin?

Line 237: Why is the MOs work only consultation? These are the most vulnerable babies and they should get the maximum care by the most senior person in the PHC.

**Monitoring and follow up**

Line 246 – 248: Who was doing the follow up and where? Why only on day 4 for FB pneumonia. Was there any follow up after they finish their treatment to know the outcome?

## **Activities of the KEMHRC RST**

Line 263 – 264: Please state how the consultation was made? And whether it was random consultation or regular for every case? If regular how often?

#### Communication

Line 278 – 280: Please mention here how frequent was this done? How many cases were assessed using this application? How accurate were they in classifying and identifying treatment? Better if you have data as this is one of the best ways of remote consultations?

Line 279 – 280: How frequent was this meeting held. How did it help in bringing changes?

**Results**

This section has important findings but needs major revision. Coverage calculation seems to be from the actual cases identified. As well all know one of the major challenges in the management of PSBI in resource limited countries like India is identification of these newborns. Hence calculation of coverage is very important. The coverage depicted here shows only coverage for identified. The key finding and informative for policy change will be identification from the expected live births. Hence you need to indicate in your study area (please look comment in the section study area description). Look also comments in each section.

Line 294 – 296: Need to rephrase it as

The public health system has successful managed 90% of the PSBI cases identified during the study period independently while 10% required consultation with the RST implying the study was implemented as planned.

#### Baseline Survey:

Based on your findings please show the referral system (How these different health facilities are linked each other. Which once are the referral sites for PSBI cases? Why the secondary level of health care was were bypassed. How are the private institutions staffed? Also describe their exact number. How few are few?

Supplemental table 2. I could not access it. Reload it or indicate where it is. At least not with the attached supplements.

Line 312 – 321: This paragraph needs to be re written clearly. Some of the data are not uniform example you said “….**about 80%-85% ASHAs were aware about danger signs in young infants and counselling for newborn care.** Then **…..** **Only 55-60% ASHAs was aware of danger signs in young infants”**

The ANMs have never identified PSBI cases before. Does this mean no NB was coming at all or how what were they doing when a new born comes to them? Clarify in order to understand.

Line 326: Mention the strength of Gentamicin available at that time?

#### Birth surveillance and postnatal visits

Do we know how much of the expected live births were identified? This is the main reason for compromising identification of PSBI cases as seen in other studies. According to the World Bank 2017 report the estimated live births in India was 18.08/1000population. Hence your area should expect 139942*0.01808/year which is equivalent to 4217 LB. You have identified only 2001. I assume that many might have delivered in the rural and district hospitals as they are relatively accessible. You may be able to estimate the proportion of the mothers who had follow in these hospitals and proportion missed taking the local circumstances. Taking this into consideration you can estimate the coverage otherwise it does not give sense calculating from the identified. Infact it should have been 100% if you calculate from the identified. I feel this has also affected your coverage of PSBI. I recommend you to give justifications for this.

Table 1: Title Follow the guideline given by PLOS.

Either you can omit this table and describe or better if you add coverage from expected.

#### Identification and referral of sick young infants

This section requires major revision

Your result mainly focuses on treatment not on identification. If I am correct WHO needed this implementation research mainly how to identify young infants with PSBI and then provision of adequate treatment? Your result in terms of provision of treatment is very nice. As I can see from your main aim of the research as well is focusing only on treatment. I was expecting coverage as well. I don’t think the number of PSBI cases expected was only 200 in 20 months in this district. If you correct the expected live births then you will get the answer. I am thinking many PSBI cases have gone directly to the private clinics and government hospitals. Is it possible to get this data or at least put estimation? Unless we have that data it will be difficult to accept 87.5% coverage of identification in this catchment.

The other issue is that in your description it seems that FB pneumonia in young infant 7 – 59 days is not considered as PSBI. Hence this part needs to be re written.

Lines 351 – 354. You have young infants sent to the sub centers. Can you separate those sent to the sub centers and to the PHC and then describe how they were managed and/or referred.

The same is true as to who evaluated them MOs/ ANMs. Do we know how many were evaluated by MOs and ANMs separately? Do we know the reason why all were not seen by the MOs if these are the most senior people you have in the PHCs.

Table 2. Follow guideline given by PLOS and correct the title of the table accordingly.

Line 366 – 368: Mention whether all have received pre referral treatment? If yes what pre referral treatment was given?

Line 378: Correct as 175 not 185.

Line 380: WHO doesn’t recommend very low birth weight to be managed as PSBI at community level and it was one of the exclusion criteria in the AFRINEST and SATT trials. Does your implementation include VLBW?

Line 382: Which one is your tertiary referral level? Is it the district hospital? If that is considered the tertiary referral level please describe some where the referral site is the district hospital.

Line 383 – 384: Just wondering how he received treatment at the PHC. Was he able to take the PO medication> state the medication that he took. Give the reason why the mother refused treatment on the second day. How comfortable was the HCP to treat such babies.

Table 3. The same follow PLOS guideline

Better if you remove the column on FB pneumonia. It is misleading. It says 22 accepted referral which is 26%. These are not offered referral rather did not accept the treatment at PHC. 26% is big. Try to explain why they have declined to be treated at the PHC rather.

One young infant with FB pneumonia came to the PHC and refused both treatment and referral. It is interesting to know why he came and why he refused every offer. It is unusual; finding and better to explain if we know the reason.

#### Treatments given to sick young infants when referral was not recommended or feasible

Please separate CSI and CI who refused referral and those with FB who were treated without offering referral to make it more informative.

Line 401 – 402: Please explain why pre referral treatment was given only to 6/81 young infants. Why was it not corrected during the process of implementation?

Line 402 – 404: This is contradictory to what you have reported in those who did not accept referral. You stated that they have treated VLBW infants. If the ANMs/MOs were not trained to give Im gentamicine, who gave treatment to the one baby who refused referral for one day. Check this with line 382 – 384. This needs explanation or correction.

Line 403: Put the exact weight than saying <1.5 kg

Line 405: Does that mean he was cured without any intervention. You can leave this as there is nothing to learn from this.

Table 4. Follow PLOS guideline.

Fb only Pneumonia in 7 – 59 days: Is it 63 or 62? Check and correct.

#### Study Progress and facilitators and challenges for implementation

General comment on this issue is related to the results. Unless we get the number of young infants going directly to the rural Hospitals and district Hospitals which I feel is the case. It is very difficult to generalize that your case identification has improved. Still this section needs revision if the result you put is the same. It is expected that there are at least equal number of babies that you failed to identify considering the PSBI cases that you expect in the district. But it may be true that most of them have gone directly to the nearby hospitals or went out of the catchment. I recommend to explain this.

Fig 4. Needs rearrangement. PSBI includes all. Hence better to change the legend as ***Total PSBI***, ***CSI/CI*** and the ***FB pneumonia in 7 – 59 days***.

Then the increment is on FB pneumonia while the CSI/CI is declining. How do we explain this?

Line 441 – 444: In your IR it seems that the rural and district hospitals were by passed to a tertiary level. If no PSBI case was being seen before the IR then where were these babies seen? If they were seen in the rural and district Hospitals, this is going to be a shift from a relatively equipped health facility to a lower and less equipped facility. The activities of these hospitals were not explained. Do you have this data?

Line 445 – 446: What does 53.3% indicate? The eligible once to be referred were 90 PSBI cases and of which 90% accepted referral (line 365). Check this and keep consistency in descriptions.

***Challenges faced***

***Table 5: As in other tables.***

***Row number 3:*** Why was it a challenge if they want to go to private centers as far as they provide appropriate treatment? Do we know their capacity to consider this as a challenge? You did not show their limitations in your description of study areas. Infact they should have been included in this IR as many babies might have gone there.

**Row 4**: Do you think you have solved this challenge? Only 6 babies received prereferral treatment out of 81 not 11 by the way. (Correct this). How come you are satisfied to say you have solved this challenge? It seems it has persisted throughout the implementation processes. Give explanation

**Row 6**: During which phase was this?

**Row 7:** It is confusing. The nurses where giving gentamicin for VLBW who did not accept referral but not for pre referral. Rationale? Most countries including WHO guideline doesn’t include VLBW in these classifications. You did not mention about the difficulty of giving amoxicillin for these babies whom I feel is more difficult.

**Discussion**

**General comment**

The discussion part is well discussed but the problem lies on calculating coverage. Unless otherwise explained the coverage of identification of PSBI in this district is not satisfactory. May be the private centers and the three hospitals are taking major share. Here either include data captured from these sites or assume based on reasonable reasoning that certain amount has gone to these sites. Please re calculate the coverage for identification of PSBI. See comments in result section.

Line 492 – 495: This part is good> If you had this information that even those who came to the PHC were preferring the private facilities the you should have collected data from those who went direct to the health facility. This is important as the main objective off WHO is identifying all young infants with PSBI and make sure that they receive adequate treatment including simplified antibiotics no just getting treatment at the PHC.

Line 503 – 504: Do we know the reasons why we did not achieve our expected output which was to provide at least 80% quality treatment? Discuss some explanations

Line 507 - 509 – was this problem continued till the end or remained a problem. Explain this. If this remains to be a problem then how do you see the ownership of this programme?

Line 538 – 541: Another way of looking effectiveness of such programmers is the change in the identification and treatment coverage of young infants with PSBI in the whole district compared to the previous years other than looking in to the increment at the PHC level only. How do you see your involvement in the community training on newborn danger signs by the ASHAs? Did it bring improvement in care seeking and more and more kids are going to the other centers as well or are you bringing those who used to go to the other centers in to the PHC. If the later is the case this is not what is expected. In case you have data try to look into this as well.

**Conclusion**

Over all the conclusion is good. But please support it with further evidence. Otherwise the fact that you have demonstrated 66% adequate treatment and identification of less than <60% will not be a strong evidence.

One good conclusion that should be clearly come out of these research is your demonstration that effective management of FB pneumonia in 7 – 59 days without the need for referral. This has to be addressed and scaled up.
