# Supplementary material for: Feasibility of implementation of simplified management of young infants with possible serious bacterial infection when referral is not feasible in tribal areas of Pune district, Maharashtra, India
Source: PLoS One. 2020 Aug 24;15(8):e0236355. doi: 10.1371/journal.pone.0236355 (PMC7446882; doi:10.1371/journal.pone.0236355)
Supplement: Attachment — Submitted filename: kemhrc pune_PSBI_manuscript_3rd Feb Apr 05 0420.docx [file pone.0236355.s008.docx]

**Full title:** Simplified management of young infants with possible serious bacterial infection (PSBI)when referral is not feasible in tribal areas of Pune district, Maharashtra, India: Implementation research for demonstration of feasibility and scale up

**Short title:** Implementation research on management ofpossible serious bacterial infection (PSBI) in young infants

**AUTHORS:** Sudipto Roy^1*^, Rutuja Patil^1^, Aditi Apte^1^, Kavita Thibe^1^, Arun Dhongade^1^, Bhagawan Pawar^2^, Yasir Bin Nisar^4^, Samira Aboubaker^5^, Shamim Ahmad Qazi^5^ Rajiv Bahl^4^, Archana Patil^3^, Sanjay Juvekar^1^, Ashish Bavdekar^1^

1 – Vadu Rural Health Program, KEM Hospital Research Centre, Pune, Maharashtra, India

2 – Health Department, Zilla Parishad, Pune District, Govt of Maharashtra, India

3 – State Family Welfare Bureau, Govt of Maharashtra, India

4– Department of Maternal, Newborn, Child and Adolescent Health and Ageing, World Health Organization, Geneva

5 - Department of Maternal, Newborn, Child and Adolescent Health, World Health Organization, Geneva (Retired)

* **CORRESPONDING AUTHOR**

E-mail: [sudipto.roy@kemhrcvadu.org](mailto:sudipto.roy@kemhrcvadu.org)

**AUTHOR CONTRIBUTIONS:** SR, YN, SA, SAQ, RB, SJ and AB conceptualized the idea and developed methodology. SA, SAQ and RB acquired funding. KT, AD, BP, AP and SR were involved in investigation and project administration. RP, SJ, AP, SR and AB were involved in resources supervision and data management. AA, SR and RP were involved in data management, data curation, formal analysis, visualization of results and preparation of original draft. SJ, AB, SA, YN and SAQ critically reviewed and edited the manuscript.

# Abstract

**Introduction:** Simplified antibiotic regimens containing gentamicin and amoxicillin areeffective in treating possible serious bacterial infection (PSBI) in young infants (YI) aged 0-59 days when referral is not possible. We implemented World Health Organization (WHO)-devised PSBI management guidelines within the public health system in Pune, India and tested feasibility and determined barriers and facilitators of implementationof these PSBI management guidelines.

**Methods:** The implementation research study was jointly conducted by KEM Hospital Research Centre (KEMHRC) Pune and the public health system (PHS), Pune. The PHS was responsible for implementation of the intervention at eight tribal primary health centres(PHC) in Pune district, India, while KEMHRC was responsible for technical support including training of PHS workers, assistance in PSBI identificationand managementand documentation of the implementation strategy.

**Results:**2001 live births were identified in the study area between August 2017 and March 2019, of whichPHS medical officers and nursesidentified and managed 90 PSBI cases, of which 90% accepted referral to tertiary care centres (as recommended),along with 85 fast-breathing pneumonia cases. The coverage of treatment was 87.5%. All YIs, except six who died (two managed at PHC and four managed at tertiary centre), were successfully treated and cured.The implementation strategy demonstrated a relatively high fidelity, acceptance and intervention penetration. Lack of training and confidence of the public health staff were major challenges faced, which were resolved to a large extent through supportive supervision and re-trainings.

**Conclusion:**We demonstrated that management of PSBI in YIs is feasible to implement in a safe and effective manner at out-patient facilities in the public health system. Technical support to the health system is required to jump-start the process; however once started, a sustainable adoption of this intervention by the health system can lead to decrease in mortality and morbidity in YIs.

# INTRODUCTION

Globally 2.5 million neonatal deaths were documented in 2018, with Southern Asia having a neonatal mortality rate of 25 per 1000 live births.(1). Worldwide, neonatal sepsis, pneumonia and meningitis together result in upto 25% of all newborn deaths(2,3)with the highest incidence of neonatal sepsis (17,000/ 1,00,000 live births) being reported in India(4). Bacterial infections account for 92% of the known causes of death for the neonatal illnesses in South Asia(5).

World Health Organization (WHO) labels potential neonatal sepsis aspossible serious bacterial infections (PSBI) in young infants (0-59 days) and recommends referral to a hospital for injectable antibiotics and supportive care. Deaths can potentially be averted by timely referral to hospitals, administration of antibiotics, supportive care and close follow up. However, in resource-limited settings, especially tribal and geographically difficult terrains, such referrals and treatments are often not feasible due to limited accessibility to hospitals(6–9).In addition, the treatments offered may not be affordable and acceptable to the families of sick young infants (10,11). Feasibility of treating severe infections in neonates and young infants in low resource settingswere first demonstrated in studies conducted across India (6), Pakistan(7), Nepal(8) and Bangladesh(9). However, the methodology in most of the sites, which included use of community health workers to manage infections,were not adopted by health systems(6,8,9). Subsequently, a series of large community based, multi-centre randomized trials in African and Asian populations (known as AFRINEST[African neonatal sepsis trial]and SATT[Simplified Antibiotic Therapy for Sepsis in Young Infants]) demonstrated the effectiveness of simplified antibiotic regimens containing injectable gentamicin, procaine penicillin and amoxicillin in out-patient settings among neonates and young infants upto two months of age with signs of PSBI where referral was not feasible(12, 13, 14, 15). WHO in 2015 synthesized available evidence to update the global guidance for management of PSBI in young infants when referral is not feasible(16). The Government of India also approved a policy for management of PSBI where referral is not possible with simplified antibiotic regimen by primary health care providers(17)(18).

However, implementation of these guidelines in the public health system requires a multi-pronged approach and involves dialogue with the policy makers and program managers, understanding barriers and facilitators for implementation and providing technical support for implementation. As part of this process, implementation research was planned across several countries within the public health systemto facilitate policy adoption and implementation at scale of the guideline for management of sick young infants with PSBI where referral is not feasible. Although, Maharashtra state in India is known for a relatively well functioning public health system and a wide spread private sector health care delivery system as compared to many other states in India (19),its tribal areas still have poor health indicators including high neonatal mortality (20). Effective management of PSBI in young infants is crucial to reducing neonatal mortality and morbidity in these areas.We describe here the process of implementation and outcomes of simplified management of PSBI in young infants in a tribal population from Western Maharashtra, India.

# METHODS: THE IMPLEMENTATION PROCESS

The aims of the implementation strategy were:

- To set up a demonstration site to deliver this intervention through policy dialogue and orientation meetings with Ministry of Health and other stakeholders at state and district level
- To demonstrate the feasibility of delivering simplified antibiotic regimens to young infants with PSBI where referral is not possible within programme setting through partnership between programme implementers and researchers

The expected outcomes of the implementation strategy were

i. All first level health facilities will provide simplified outpatient management of PSBI when referral is not possible.
ii. All or at least 80% of identified sick young infants will receive treatment.
iii. All or at least 80% of the sick young infants treated will receive adequate treatment (Adequate treatment was considered as all antibiotic doses for the first two days followed by at least 3 doses of injection gentamicin and 6 doses oral amoxicillin over the next five days)

## Design

The study was part of a multi-site and multi-country implementation research coordinated by WHO to test the feasibility of implementing PSBI guidelines where referral is not feasible in a programme setting through an innovative approach of partnership between King Edward Memorial Hospital Research Centre (KEMHRC) Pune, a research institution and the public health system in Maharashtra state and specifically, Pune district. A common protocol and common tools were developed by the WHO team and used across four different sites in India. The guidelines for management of PSBI in young infants (YI), were referral is not feasible constituted the study intervention while the implementation strategy included its introduction and implementation within the public health system in the study area. We have deliberately linked the intervention and the implementation strategy and not separated them while describing the study methods, as it reflects the actual ‘real-world’ conditions in which this study was conducted.

## Study partners

The public health system, Pune district, was responsible for implementing the study activities including identification and management of PSBI in young infants, ensuring effective referral systems and availability of medicines and equipment.The team from KEMHRC was responsible for providing technical assistance, capacity development and supportive supervision to public health system workers as well as research data documentation. This clear division of responsibilities was a deliberate decision to ensure that the study could replicate programme conditions wherein the public health system is expected to implement the interventionwith limited external assistance. The public health system team is henceforth referred to as ‘Implementation Team’ (IT) and KEMHRC team is termed as ‘Research and Support Team’ (RST). Fig 1 shows the study logic framework for this study in the form of inputs, activities and outcomes by both study partners.

***Fig 1:Logical framework for an implementation strategy for introducing PSBI management in young infants in the public health system, Pune.***

## Policy dialogue for implementing adapted guidelines

A national level consultative meeting was held in 2016 to review, adopt and adapt the WHO guidelines for PSBI management which resulted in certain changes in the Indian IMNCI guidelines(Supplementary table 1). These changes included consolidation of signs and symptoms of PSBI based on which PSBI sub-classifications were modified and increased dose of oral amoxicillin was added. These changes were based on the evidence generated from the AFRINEST and SATT studies (13, 14, 15). A policy decision was also made to adopt a regimen of seven-day injectable gentamicin and oral amoxicillin for PSBI when referral was not feasible. The meeting resulted in a consensus on adaptation and implementation in four demonstration sites in selected states in India.

A second orientation and policy dialogue was conducted at the Maharashtra State level in early 2017 by study investigators from KEMHRC to ensure the buy-in from local health authorities and decision makers including Additional Director Health Services, State of Maharashtra (ADHS) and the District Health Officer (DHO), Pune. The public health system agreed to be a partner in the study and selection of the study area was decided at this meeting. This dialogue resulted in the establishment of a Technical Support Unit (TSU) with representation from both public health system and technical experts from KEMHRC. It comprised of senior public health officials from public health system, a paediatrician of government medical college and the investigators representing the RST. The role of the TSU was to provide guidance and leadership to the project and review the progress of the project and provide suggestions to challenges arising during the study conduct.The public health system personnel were involved in the development of study implementation plan and also in its implementation. Anorder, directing health workers to implement the adapted PSBI guidelines, was issued from the district health officials to all health facilities involved in the study.

## Ethical considerations

The study was initiated after obtaining approval from KEMHRC and the WHO research ethics review committee. The study was implemented following good clinical practices and Indian Council of Medical Research guidelines for research involving human participants.

## Study area

The study was conducted across eight tribal Primary Health Centres (PHCs) situated in three blocks of Pune district namely Junnar, Ambegaon and Khed (**Fig 2**). All the selected villages in the study area were located in geographically difficult-to-access terrains but had public health infrastructure available for implementation. These PHCs are located in the Western Ghats of Maharashtra, covering total 1,40,000 population. Each PHC catered to around 20,000 population and comprised of 5-12 sub-centres (SC) serving about 3,000 population each.

***Fig 2: Map of the study area in Pune district***

## Baseline survey and mapping of referral system

A baseline survey was conducted by the RST in the eight selected PHCs and 15 randomly selected health sub-centres within these PHCs in order to asses readiness of facilities to implement the PSBI guidelines. This included review and availability of staff, knowledge, training and practice of ANMs, equipment, medicines, consumables and reporting systems relevant to PSBI management. The survey also included semi-structured interviews administered to 62 community health workers known as Accredited Social Health Activists (ASHA) and 121 mothers of young infants to understand their knowledge and practices related to care of young infants. Additionally, mapping was done of public as well as private health facilities with Neonatal Intensive Care Units (NICU)/ Special Newborn Care Units (SNCU) in increasing order of distance from the PHCs.The availability of ambulances in working conditions and the existing process of referrals were also documented.

## IT team activities

#### Trainings and orientation

Officials from the public health system and a member of the RST were trained as “master trainers” by trainers from WHO and IMNCI program of India in an extensive three-day training on assessment, classification and management of PSBI along with newborn care. The training included interactive class room training as well as hands on clinical practice. The algorithm for identification and management of PSBI was adopted from the WHO PSBI guidelines (21). The master trainers then used the same training methods and materials to train all medical officers (MO) and Auxiliary Nurse Midwives (ANMs) in the study area. The MOs with support from RST,oriented ASHAs on identifying danger signs of PSBI and essential newborn care, at their respective PHCs. Twelve MOs, 68 ANMs and 265 ASHAs in the eight selected PHCs were trained through these sessions.

#### Implementation of simplified management of sick young infants [**Fig 3**]

i. Identification of sick young infants

The ANMs, as part of their routine activities recorded all pregnancies and births in their coverage area and ASHAs visited all neonates on days 1, 3, 7, 14, 30 and 42 of birth as per national guidelines. During these visits, ASHAs counselled the caregivers about danger signs of PSBI in addition to providing newborn care and identifying danger signs of illness if they came across a sick young infant. YIs with axillary temperature 37.5^o^C or above, axillary temperature less than 35.5^o^C, fast breathing, chest indrawing, convulsions, difficulty in feeding or reduced movements were then brought to ANMs or MOs for further assessment and intervention. ASHAs were instructed to contact ANMs first, either in-person at SC or telephonicallyand ANMs would assess YIs either at SCs or PHCs. If ANMs were unavailable or the PHC was nearer, ASHAs would bring sick YIs to the MOs at PHCs for assessment. At PHC/SC level, the sick young infants were assessed by MOs or ANMs for signs of PSBI and referred to a higher facility, if needed. The sick young infants were classified aseitherPSBI, with sub-classifications of clinical severe infection (CSI), critical illness (CI) and fast breathing (FB) in 0-6 days old infants,or FB in 7-59 days old infants(21).

ii. Referral to tertiary health care facilities

As per guidelines, caregivers of young infantsclassified as CSI, CI or FB (only in 0-6 days old) were counselled about the need for hospitalization and were advised referralby MOs and ANMs to the tertiary level health facility for treatment. In many cases, ASHAs accompanied the young infants to the tertiary care.Such young infants were given first dose of gentamicin intramuscularly before referral to tertiary health care facilities. For every referral, a referral slip was handed over to the parent to show to the tertiary care facility.The caregivers were given instructions to maintain warmth and provide breastfeeding to the baby during transportation. In infants classified as CI, CSI or FB (only in 0-6 days old), whenever the referral was not feasible, MO/ANM continued to provide care as per adapted PSBI management algorithm. (21)

iii. Treatment when referral is not feasible or referral is not recommended

When referral was not feasible, even after making all efforts to counsel and refer, infants aged 0-6 days with FB and young infants with any sign of CSIor CI were treated with a course of intramuscular gentamicin 5-7.5 mg/kg once daily for 7 days and oral amoxicillin 50mg/kg twice daily for 7 days on an outpatient basis. All doses of injection gentamicin and first daily dose of oral amoxicillin were administered by the ANM in consultation with the MO at the PHC and the second oral amoxicillin dose for the day was administered by mother after being taught to ascertain the correct dose and method of administration by ANM. All young infants aged 7-59 days with FB were treated with oral amoxicillin 50mg/kg twice daily for 7 days as per the treatment algorithm (21). Caregivers were taught to administer oral amoxicillin at home, counselled on identifying signs of deterioration and were asked to bring the infant back to the ANM if there was any such sign. If the child`s health would deteriorate, they were again advised referral.

iv. Monitoring and follow up

Young infants with any sign of CSI or CI, or FB (only in 0-6 days old infants)who received outpatient treatment were followed up every day till day 7 of treatment, while infants aged 7-59 days with only FB who received oral amoxicillin were followed up at least once on the 4^th^day of treatment by ASHA/ANMs to assess the infant’s condition and adherence to treatment. In case of referred sick young infants, first telephonic follow up was done by ANM during hospitalisation and second follow up was done at home by ANM with ASHA after hospital discharge within 14 days of onset. Assessments performed by ASHAs during home visits, including identification of danger signs and subsequent visit to ANMs/MOs were recorded in the Home-based neonatal care (HBNC) cards and the clinical assessment, identification and management of PSBI by ANMs and MOs were recorded in IMNCI forms at PHCs.

***Fig 3.Flow chart of study activities. Fig 3 shows the process of identification, assessment, classification and treatment of PSBI in the study***

## Activities of the KEMHRC RST

The RST, in consultation with the IT, developed the study protocol.The RST also developed the study tools. Itorganized and conducted training for all health workers in the study area. The RST did not participate in any implementation activities but was available for on-field support to the health care workers and whenever needed provided additional training related toassessment, classification and management of PSBI cases.Study activities were jointly supervised by members of the RST and senior district level public health authorities. RST members were occasionally consulted by MOs/ANMs regarding identification of PSBI and use of the treatment algorithms for appropriate management. The RST would coordinate with the block and district level authorities to solve challenges and problems in the field. The RST transcribed data related to PSBI case management from records maintained by healthcare workers onto case record forms developed for this study. Thus, source documents were completed by the health care workers and the case record forms were designed to transcribe data for pregnancy and birth surveillance, HBNC home visits, classification of sick young infants, treatment and referral details and facility as well as home-based follow up from source documents. Finally, the RST was responsible for analysis and presentation of results from this study.

#### Communication

Based on the results of the baseline survey, information, education and communication tools were developed by the RST with inputs from the health workers. This consisted of charts and booklets depicting signs and symptoms of PSBI and their management as per adapted study guidelines when the referral is not feasible. The RST communicated with theIT during their weekly meetings at the respective PHCs in addition to the frequent face to face and telephonic interactions during and after identification of the suspected PSBI cases. The RST andImplementation Team also used multimedia technology like WhatsApp videos to confirm the signs in young infants with suspected PSBI. In addition, there were regular meetings with block and district level authorities to discuss the challenges faced and devise solutions to overcome these.

#### Data Management and Analysis

Data was entered into electronic record forms created using REDcap software. The data management was done on local standalone server with back up facilities. Anonymized dataset was extracted and was used for analysis for quality control. Descriptive analysis was done using Microsoft Excel calculations of frequencies and proportions.

## Study period

The study began in March 2017 and ended in August 2019. The baseline and preparatory activities were conducted between March 2017 and July 2017 while field activities were conducted between August 2017 and March 2019.

# RESULTS

The study was largely implemented as planned, with the public health system identifying and managing about 90% PSBI cases independently, while in about 10% cases, public health workers consulted the RST about appropriate course of action.

## Preparatory phase

#### Baseline Survey:

The baseline survey of the facilities in the study area showed thatall the eight PHCs and 64 SCs in the study area were functional in terms of availability of human resources, medicines and supplies, updated documentation and patient flow. There arethree30-bedded rural hospitals (one in each block) located within 20-40 km distance from each PHC whereas a 100-bedded subdistrict hospital is located at 70-80 km from the PHCs. The rural hospitals (similar to community health centres elsewhere in India) offer secondary health care with availability of pediatrician and inpatient facilities but do not have intensive care unit. The subdistrict hospital has a pediatrician but no neonatal intensive care facility. There are few private health facilities, within the study area, with some having intensive care units. All PHCs were equipped with one ambulance each for referral to a higher health facility. However, vehicles were not available at all times and patientssometimes needed to spend money for fuel or arrange for alternate transport to reach a higher health facility. There were adequate human resources working at the eight PHCs. The details of the staff and population catered for the eight PHCs in given in supplementary table 2.

We found that 95% ASHAs reported that they conduct home visits post-delivery as per government guidelines and about 80%-85% ASHAs were aware about danger signs in young infants and counselling for newborn care. Amongst the caregivers, 90% mothers had received counselling on care of young infants post-delivery. Only 55-60% ASHAs were aware of danger signs in young infants and only 50% of the mothers received counselling on the same. None of the ANMs reported to have identified and managed PSBI in young infants in the past three months. A crucial reason cited was lack of confidence and fear of mishap in handling sick young infants at a peripheral health facility. However, they showed readiness to manage cases with PSBI if they were provided with appropriate training and technical support. This survey also revealed that caregivers of young infants from the tribal population generally did not show readiness for accepting referral and preferred home-based care due to cultural and socioeconomic reasons (lack of belief in healthcare, non-affordability, unavailability of transport, loss of wages). Most of the ANMs were not experienced in administration of intramuscular gentamicin in infants. Most of the PHCs were equipped with baby warmer but were not adequately equipped with weighing scales, thermometers and stopwatches or timers to count respiratory rate. Oral amoxicillin was not available at all centres at the time of the survey, whereas injectable gentamicin and syringes for intramuscular administration were available.

#### Discussion at State and district level for assurance of supplies

Observations from the baseline assessment were communicated by RST to the senior officials from the public health system in face to face meetings. Assurance for support was obtained from the government officials in order to maintain the health staff and adequate supplies of amoxicillin, gentamicin and consumables. Before implementation, the PHCs and SCs were strengthened with provision of weighing scales, thermometers and stopwatches/timers for counting respiratory rate to all ASHAs.

## Implementation phase

#### Birth surveillance and postnatal visits

During the study period of August 2017 to March 2019, 2001 live births were recorded by the IT and post-natal home visits were made by ASHAs in majority of the cases (**Table 1**). A decrease from the first post-natal visit in subsequent postnatal visits were due to parents moving out of study area after discharge from the health facility and lack of documentation by the ASHAs for some of the visits.

**Table 1: Birth surveillance and postnatal visits (July 2017 to March 2019)**

| **Birth surveillance** | **Total number** |
| --- | --- |
| Population of catchment area | 139942 |
| Total live births identified by public health system | 2001 |
| **Post-natal visits by day by ASHAs, N(% of live births)** | |
| No of postnatal visits on Day 1 | 1777(88.8) |
| No of postnatal visits on Day 3 | Not available |
| No of postnatal visits on Day 7 | 1316(65.7) |
| No of postnatal visits on Day 14 | 1420(70.9) |
| No of postnatal visits on Day 21 | 1400(69.9) |
| No of postnatal visits on Day 28 | 1382(69.0) |
| No of postnatal visits on Day 42 | 1380(68.9) |
| No of postnatal visits on Day 59 | 1360(67.9) |

***Table 1 shows the total number of births and post-natal visits conducted by ASHAs in the study area during the study period***

#### Identification and referral of sick young infants

A total of 545 young infants were identified during the study implementation phase, of which 90 had signs of PSBI (46 CSI, 34 CI and 10 FB in 0-6 days old infants) and 85 infants aged 7-59 days had only FB. [Table 2]. Assuming a 10% incidence (13, 14, 15) of PSBI among all live births, the expected number of young infants with any sign of PSBI or FB in 7-59 days old would be 200, whereas in this study, 175 young infants with PSBI or FB in 7-59 days old were identified and treated,resulting in a coverage of 87.5% (Table 2).

*Identification of illness:* Of the total number of young infants with signs and symptoms of PSBI, almost similar proportion of cases were brought by families to PHC/SC, referred by ASHAs to PHC/SC and identified by MO/ANM following birth or during immunisation session.Approximately 85% of the sick young infants aged 7-59 days with FB were either brought by families or referred by ASHAs [**Table 2**].

**Table 2: Identification of sick young infants during study duration**

| **Parameters** | **All sick young infants**  **(N=175)** | **PSBI (N = 90)** | | | **Fast breathing (FB)**  **in 7-59 days**  **(N=85)** |
| --- | --- | --- | --- | --- | --- |
|  |  | **Clinical severe infection (CSI) (N=46)** | **Critical illness (CI)**  **(N=34)** | **Fast breathing (FB) in 0-6 days**  **(N=10)** |  |
| **Identification of sick young infants, n (%)** | | | | | |
| Brought by families to PHC/SC | 67 (38.3) | 20 (43.5) | 5 (14.7) | 4(40.0) | 38 (44.7) |
| Identified by ASHAs in the community and referred to PHC/SC | 62 (35.4) | 15 (32.6) | 11 (32.3) | 1(10.0) | 35 (41.2) |
| Identified at PHC/SC by ANM/MO following birth or during immunisation sessions | 46 (26.3) | 11 (23.9) | 18 (52.9) | 5(50.0) | 12 (14.1) |

***Table 2 shows the place of and person who first identifiedsigns and symptoms of PSBI in the study***

*Referral to government or private tertiary care and management of sick young infant at PHC/SC:* Among the 90PSBI cases that were eligible for referral and were advised referral to a government tertiary care centre, 81 (90%) accepted referral. Among 81 young infants with any sign of PSBI who accepted referral, 48 (59.3%) were hospitalized at a government tertiary care, while33 (40.7%) went to visit a private facility.Those who refused referral (n=9) received simplified antibiotic treatment on outpatient basis at PHC/SC level, of which five were classified as CSI, three as fast breathing in infants aged 0-6 days and one had CI. One infant had CI and was managed at the PHC [**Table 3].**In case of FB in infants aged 7-59 days, while all 85 such cases were offered treatment at PHCs by MO/ANM, 62 (72.9%) infants were treated at PHC/SC with oral amoxicillin, while 22 (25.9%) infants refused treatment at PHC/SC and went to visit a private facility.One case of FB in 7-59 days old infant, which refused treatment migrated out of the study area and could not be followed up for outcome. While we did not collect information on treatment given at private health facilities, we expect that the common treatments given there for sepsis and pneumonia in young infants includeinjectable or oral amoxicillin clavulanate, third generation cephalosporins and macrolide antibiotics.

*Clinical outcomes in sick young infants:* Ninety-six percent of all 185 infants were cured of their illnesses, with similar cure rates atpublic and private healthcare centres. Six (five CSI and one CI) out of 90(6.6%) young infants with PSBI died. [**Table 3**], all of whom were very low birth weight [average birth weight 1.4 ± 0.21 kg] and pre-term (less than 34 weeks gestational age) and all were diagnosed with signs of PSBI on day 0 or 1 of life. All of these infants were referred to the district hospital but only four accepted the referral (including one self-referral to private health care). Two infants refused referral and were started on treatment at the PHC on day-1 of identification and stopped treatment against medical advice on day-2.

Among infants aged 7-59 days with FB, treatment success was 100%, irrespective of the place of treatment.

**Table 3: Referral, treatment and outcome of sick young infants during study duration**

| **Referral and treatment of sick young infants, n (%)** | | | | | |
| --- | --- | --- | --- | --- | --- |
|  | **PSBI (N = 90)** | **Clinical severe infection (N=46)** | **Critical illness (CI)**  **(N=34)** | **Fast breathing in 0-6 days**  **(N=10)** | **Fast breathing**  **in 7-59 days**  **(N=85)** |
| Referral to tertiary government facility accepted | 48 (53.3) | 17 (37.0) | 27 (79.4) | 4(40.0) | N.A. |
| Referral was accepted but went to private facility | 33 (36.6) | 24 (52.2) | 06 (17.6) | 3(30.0) | 22 (25.9) |
| Refused referral/referral not feasible and accepted treatment at PHC/SC | 9 (10) | 5 (10.9) | 1 (02.9) | 3(30.0) | N.A. |
| Referral not recommended and treated at PHC/SC | N.A. | N.A. | N.A. | N.A. | 62 (72.9) |
| Refused referral and also refused treatment at PHC/SC | 0 (0.0) | 0(0.0) | 0 (0.0) | 0 (0.0) | 1 (1.2) |
| **Follow up of all sick young infants,n (%)** | | | | | |
|  | **All infection cases (N = 175)** | **Clinical severe infection (N=46)** | **Critical illness (CI)**  **(N=34)** | **Fast breathing in 0-6 days**  **(N=10)** | **Fast breathing**  **in 7-59 days**  **(N=85)** |
| Completion of at least one follow-up visit by ANM ^#^ | 174 (99.4) | 46 (100.0) | 34 (100.0) | 10(100.0) | 84 (98.8) |
| **Final treatment outcomes, n (%)** | | | | | |
|  | **All infection cases (N = 175)** | **Clinical severe infection (N=46)** | **Critical illness (CI)**  **(N=34)** | **Fast breathing in 0-6 days**  **(N=10)** | **Fast breathing**  **in 7-59 days**  **(N=85)** |
| Clinical treatment success^$^ | 168 (96.0) | 41 (89.1) | 33 (97.1) | 10 (100.0) | 84 (98.8) |
| Death | 6 (03.4) | 5 (10.9) | 1 (02.9) | 0 (0.0) | 0 (0.0) |
| Outcome unknown | 1 (0.6) | 0 (0.0) | 0 (0.0) | 0 (0.0) | 1 (1.2) |

***Table 3 has three parts: Referral and treatment of YIs with PSBI, follow-up of all these YIs by public health system staff and final treatment outcomes for these YIs.***

***# - follow up was completed within 8 days for sick young infants treatedat PHC and telephonic follow up for infants referred and hospitalised at tertiary centre/private facility followed by home visits by ANM after discharge; $ - The treatments include treatments given at the tertiary referral as well as simplified regimens offered at PHC/SC.***

***N.A.: Not applicable***

#### Treatments given to sick young infants when referral was not recommended or feasible

Nine sick infants with PSBI(5 CSI, 1 CI, 3 FB in 0-6 days old infants) and 62 infants aged 7-59 days with FB pneumonia received simplified regimen at PHC/SC [**Table 4**]. Sixty-seven percent of the PSBI cases received complete treatment with gentamicin and oral amoxicillin whereas two sick young infants with CSI received one injection of gentamicin and three days of oral amoxicillin. Pre-referral doses of gentamicin were given in only 7.1% (6 out of 84) PSBI before referral to higher centres. One infant did not receive any treatment as its weight was less than 1.5 kg and MOs/ANMs were not trained to treat such very low birth weight babies. Since its parents refused referral, the infant was offered Kangaroo mother care and closely followed up and went on to recover from the illness.

Out of 62 FB cases in 7-59 days age group who were treated at PHC/SC level,87%(n=55) completed treatment with oral amoxicillin and 9.52% (n=6) infants received less than adequate treatment for 6-13 days.

Of two sick young infants who were treated at PHC and died, one received two doses of oral amoxicillin and no injection gentamicin and the other one received three days treatment with injection gentamicin and oral amoxicillin. Both were discharged against medical advice and subsequent deaths (on day three and six respectively) were reported by ASHAs following home visits.

***Table 4: Treatment for sick young infants when referral was not recommended or feasible***

| **Parameters** | **All PSBI cases (N=9)** | **Clinical severe infection (N=5)** | **Critical illness (CI)**  **(N=1)** | **Fast breathing in 0-6 days**  **(N=3)** | **Fast breathing**  **in 7-59 days**  **(N=63)** |
| --- | --- | --- | --- | --- | --- |
| **Intramuscular gentamicin doses (5-7.5 mg/kg once daily), n(%)** | | | | | |
| Received all 7 injections | 6 (66.7) | 2 (40.00) | 1 (100.0) | 3(100.0) | NA |
| Received 3 injections* | 1 (11.1) | 1 (20.0) | 0 (0.0) | 0(0.0) | NA |
| Received 1 injections* | 1(11.1) | 1 (20.0) | 0(0.0) | 0(0.0) | NA |
| Received no injection^#^ | 1(11.1) | 1(20.0) | -- | -- | NA |
| **Oral Amoxicillin doses (50mg/kg twice daily), n(%)** | | | | | |
| Received all 14 days of treatment | 61(84.7) | 2(40.0) | 1(100.0) | 3(100.0) | 55(87.3) |
| Received 10-13 days of treatment | 5 (6.9) | 0(0.0) | 00(0.0) | 0(0.0) | 5(7.93) |
| Received 6-9 days of treatment* | 1 (1.4) | 0(0.0) | 0(0.0) | 0(0.0) | 1(1.59) |
| Received ≤5 days of treatment | 4 (5.6) | 2(40.0) | 0(0.0) | 0(0.0) | 1(1.59) |
| Received no amoxicillin^#^ | 1 (1.4) | 1(20.0) | -- | -- | 0(0.0) |

***Table 4 describes treatment given by public health staff on out-patient basis. This includes only fast-breathing pneumonia for which out-patient treatment with amoxicillin is the recommended guideline as well as PSBI cases where referral was refused or not possible.***

***# The infant did not receive any treatment as body weight was less than 1.5 kg; * - outcome is death before completion of 7 days***

An important finding from this study was the relative safety of the interventions as well as the implementation strategy, with none of the patients reporting adverse events.

#### Study Progress and facilitators andchallenges for implementation

***Study progress****:* The number of cases managed in the first five months were less than expected, with few health staff identifying and managing PSBI cases. The RST provided on-field support to all health staff while public health officials, through their feedback mechanisms, encouraged health staff to identify and manage PSBI cases. With these efforts, case reporting did increase over the remaining 15 months of the study period (**Fig 4**).

***Figure 4: Progression of identification of sick young infants with PSBI. Fig 4 shows the number of PSBI and fast-breathing pneumonia cases identified separately in blocks of five-month periods over the study period.***

***Facilitators for success:***The ownership of the study by the public health officials at the state and district levels and establishment of TSU were major facilitators that contributed to a smooth implementation of the study. This buy-in of the public health system was a crucial aspect of this implementation research study in terms of potential sustainability and scale-up.

Training sessions for all health staff in the study areas played a major role in enhancing the knowledge and capacity building of MOs, ANMs and ASHAs in identification and management of the PSBI cases. This also provided opportunity for trainers to allay their apprehension and lack of confidence about handling sick young infants. Study-related communication at all levels in public health system increased awareness about the PSBI and importance of treatment and timely referral in these cases amongst the IT. This helped the IT in prioritising management of sick young infants through simplified regimes and increased their ownership towards the program. These was evident from the change in practices regarding managing PSBI in young infants, with the baseline survey showing that no PSBI case was managed at PHCs prior to the study implementation.

A robust and effective referral system ensured that a majority (53.3%) of young infants with PSBI, who were eligible for referral, to tertiary care health centres, actually accepted referral to such centres. The role of ASHAs as specifically important in persuading the caregivers to accept the referrals. In some cases, ASHAs, being the member of the same community, with help from the local community leaders and village heads also arranged for logistics or monetary support from within the village for transport of the sick young infants to health care facility when ambulance was not available immediately.

***Challenges faced:***Our study faced various challenges and barriers during implementation, which resulted in numerous modifications of the predefined protocol (**Table 5**).

***Table 5: Challenges face during implementation research, steps taken and end result***

| **No** | **Issue** | **Steps taken** | **End result** |
| --- | --- | --- | --- |
| 1 | Inadequate case reporting in the first six months and lack of coordination between ASHAs and ANMs | Re orientation of ANM and ASHAs in identifying PSBI | Increased case reporting in next 15 months period of the study. However, few ASHAs and ANMs still reported fewer cases/population than others |
|  |  | Counselling of ANMs by MOs to increase case-finding efforts |  |
|  |  | On-field support for low-performing ASHAs and ANMs |  |
| 2 | Documentation by ANMs/MOs | Re-orientation sessions and hand-holding for recording complete data for individual cases. | Most ANMs recorded adequately; ANMs in busier PHCs complained of lack of adequate time to fill in IMNCI forms completely |
| 3 | Parents taking their sick YI to private providers in-spite of advice for referral to government tertiary care centres | MOs and ANMs counselled parents for referral to government tertiary care centers | Referrals to Government facilities improved over the study period, but few parents still preferred private providers |
| 4 | A smaller proportion of PSBI cases received pre-referral doses. | Discussed with relevant MOs to support ANMs in administering pre-referral Gentamicin injections | The number improved, but only 11 PSBI cases received pre-referral doses in the study |
| 5 | Unavailability of ambulance service needed for prompt transport of sick young infant to referral centre | ASHAs and ANMs coordinated with the village heads for making a vehicle available for the patient.  District health authorities ensured availability of ambulance at all PHCs. | The sick young infant got access to treatment at tertiary care centre. |
| 6 | Unavailability of antibiotics in some PHC/SCs | The RST coordinated between different health facilities to make the supplies available to the ones which did not have enough supply. District health authorities ensured availability of medicines at all PHCs | The antibiotics were subsequently made available through the study period |
| 7 | Lack of experience in administration of gentamicin injection in low birth weight infants | Retraining and confidence building was done by the SRT | The MO/ANM did not administer the prereferral dose in spite of the trainings as they were afraid of being responsible in case of mishap |

***Table 5 shows the different challenges faced in the implementation process and steps taken to solve them***

# DISCUSSION

Broadly speaking, this implementation research shows that it is feasible to implement the strategy of early identification, timely referral and simplified management of sick young infants inthe public health system in tribal areas of Maharashtra. The most crucial factor for successful implementation was the complete ownership by the public health system, especially at the state level. The involvement of senior leadership in the public health system also ensures that the intervention can be scaled-up to other parts of the district and the state and can possibly be sustained in the form of a programme, though further policy-related dialogues are needed for this. Secondly, technical support to the public health system for an initial period of time helps to resolve many issues and streamline the implementation process.

One of the most important outcomes of this study was that fast breathing pneumonia in 7-59 days young infants can be effectively and safely treated with oral amoxicillin on an out-patient basis without referral in a programme setting as per current PSBI guideline recommendations. Almost 73% of such infants were successfully treated by MOs/ANMs and were completely cured, with 87% babies receiving full treatment regimen and 97% of the babies receiving more than 5 days treatment with amoxicillin. The rest (27%) preferred to visit private healthcare centres where they could have received treatment with other antibiotics and were also completely cured. This finding enables the public health system to effectively triage such cases and avoid referring them unnecessarily to tertiary care centres, thus reducing the workload at these centres as well as reducing the exposure of these young infants to higher-level anti-microbials. The current Government of India guidelines recommend treatment by injectable gentamicin and oral amoxicillin for fast-breathing pneumonias in all young infants aged 7-59 days. Our study data can help to inform the guidelines for necessary revision.

A relatively low case fatality rate of 3.4% (six deaths out of 175 cases) show that the adapted PSBI guidelines can help to reduce neonatal and infant mortality, if implemented appropriately by the public health system.

We had a very high proportion of treatment coverage, which was a result of effective and timely coordination between different facilities and cadre of the public health system. The processes conducted in our study can again inform the successful adoption of these guidelines in other districts and states in India, subject to local modifications.

A striking finding from our study was the high acceptance (90%) of referral to tertiary care centres among eligible infants with PSBI. While all eligible PSBI infants were advised referral to government tertiary care centres, a substantial proportion (about 36%) preferred to visit a private tertiary care. The high number of referrals to government tertiary care hospitals was due to presence of effective communication amongst the care-providers and increased awareness and an effective referral system. Further, linkages with the tertiary government healthcare centres, facilitated through senior state level health officials, helped in successful referrals. While not assessed in details, on interviewing some parents, we found that the major reason for referral to private facilities was easy acceptability, familiarity with the private physicians and proximity of the private facility as compared to a public facility. We thus recommend encouraging partnership between the public and private sector for healthcare delivery and implementing the guidelines for PSBI management, considering that one third of sick infants were managed at the private healthcare facilities were perceived to be more accessible to the general population than government tertiary care unit. However, quality, cost and completeness of treatment in private care may not be optimal and may promote irrational use of antibiotics. In this regard, bringing-in of the private sector in implementing the guidelines will encourage standardized approach and ensure appropriate treatment for PSBI management.

Only about 10% of the PSBI cases who were recommended referral, refused referral and were administered simplified management at PHC/SC level. The common reasons for refusal of referral were lack of manpower and family support, financial issues and lack of transportation of facilities. We need to further explore ways to facilitate referrals for such cases, since the recommended guidelines for PSBI cases is treatment at a tertiary care facility. The quality of treatment received at PHC level was optimal for almost 66% of the PSBI cases in those who accepted the treatment.

Readiness of the public health system:We found that all health staff, including MOs, ANMs and ASHAs needed re-training in IMNCI including PSBI management. There were no major challenges regarding availability of medicines and consumables, however, equipment such as accurate weighing machines, stopwatches/respiratory rate timers needed for managing PSBI was inadequate. These issues, though can be relatively easily resolved within the public health system, need to be assessed before commencing implementation.

Acceptability and implementation penetration:Within the public health system, the study strategy was generally well accepted. However, our study showed that initially, facilitation and hand-holding for a limited time, built confidence of entire public health system; especially the primary healthcare workers. This was reflected in an increase in the number of cases identified and managed after as the study progressed. The hesitancy of ANMs to administer injectable gentamicin in young infants due to fear of adverse effects was minimized with refresher training and constant on-field support. The awareness about management of sick young infants and feeling of responsibility increased amongst Implementation Team, due to which the MOs/ANMs who did not manage a single sick infant with PSBI at baseline, could effectively manage majority sick infants at PHC/SCs when referral was not feasible. The acceptance amongst public health system and top-down communication was found to be a major facilitator. Almost all health staff of the public health systemincluding MOs, ANMs and ASHAs readily accepted and delivered the intervention, but needed technical support and hand-holding as this was being done for the first time in the public health system.

Fidelity and System adaptations required:In PSBI cases, with 90% young infants successfully referred, though only 53% accepted referral to government tertiary facilities as recommended, we can say that fidelity was largely achieved, while, among those not referred, further efforts need to be taken to ensure that all such infants are adequately treated on an out-patient basis. These efforts could include more effective counselling of care-givers, greater involvement of local community leaders in counselling efforts and more support from senior public health officials.No major system adaptations were required. The low proportion of pre-referral injectable gentamicin in this study indicates that there is a need for motivation and confidence-building,including support of senior public health authorities, of MOs, ANMs and ASHAs to administer pre-referral gentamicin.

A cluster randomized study in Ethiopia on effect on neonatal mortality of newborn infectionmanagement at health posts when referral is not possible reported in 2017 that when referral to higher levels of care is not possible, community health workers can deliver outpatient antibiotic treatment of newborn infants with PSBI, though estimated treatment coverage in a rural Ethiopian setting was only around 50%^22^.

This implementation science research was appreciated by the public health system and in future, the RST was invited to continue working with the public health system.The TSU and the public health system is keen to scale up these adapted PSBI guidelines to entire state of Maharashtra. This will be done by scaling up the training activities and awareness program through the district training teams in the state. However, in order to make the program sustainable, it is important that the role of RST is taken over by the public health system and TSU.

# CONCLUSION

The study demonstrated that the identification and simplified management of PSBI and pneumonia in young infants can be implemented in a safe and effective manner at out-patient facilities in the public health system when referral is not feasible. Technical support to the health system is required to jump-start the process; however once started, a sustainable adoption of this intervention by the health system can lead to decrease in mortality and morbidity in young infants.

# ACKNOWLEDGEMENTS

- Dr. Makarand Ghorpade, study coordinator, KEM Hospital Research Centre Pune for helping to coordinate study activities
- Technical Support Unit members including Dr. Arti Kinikar, Dr. Sanjeevkumar Jadhav and Dr. Ashish Bharti for providing technical overview
- Taluka Health Officers of Junnar, Ambegaon and Khed blocks, Health Department, Zilla Parishad, Pune for supervising PHC staff
- MOs, ANMs and ASHAs of all eight study PHCs, Health Department, Zilla Parishad, Pune for implementing PSBI guidelines
- Dr. Harish Chellani; Dr. Sugandha Arya, Indian national IMNCI master trainers for conducting the study training
- Field Research Assistants and clinical coordinators of KEMHRC team for supporting the PHC staff
- Kaushik Ghosh, developer of data management system in Redcap

# REFERENCES

1.	UN Inter-agency Group for Child Mortality Estimation. Levels and trends in Child Mortality. UNICEF, New York. 2019.

2.	Chan GJ, Lee ACC, Baqui AH, Tan J, Black RE. Risk of early-onset neonatal infection with maternal infection or colonization: a global systematic review and meta-analysis. PLoS medicine. 2013;10:e1001502.

3.	Liu L, Oza S, Hogan D, Chu Y, Perin J, Zhu J, et al. Global, regional, and national causes of under-5 mortality in 2000–15: an updated systematic analysis with implications for the Sustainable Development Goals. The Lancet. 2016;388:3027–35.

4.	Fleischmann-Struzek C, Goldfarb DM, Schlattmann P, Schlapbach LJ, Reinhart K, Kissoon N. The global burden of paediatric and neonatal sepsis: a systematic review. The Lancet Respiratory medicine. 2018; 6:223–30.

5.	Saha SK, Schrag SJ, El Arifeen S, Mullany LC, Shahidul Islam M, Shang N, et al. Causes and incidence of community-acquired serious infections among young children in south Asia (ANISA): an observational cohort study. The Lancet. 2018;392:145–59.

6.	Bang AT, Bang RA, Baitule SB, Reddy MH, Deshmukh MDD. Effect of home-based neonatal care and management of sepsis on neonatal mortality: Field trial in rural India. Lancet. 1999;354:1955–61.

7.	Zaidi AKM, Tikmani SS, Warraich HJ, Darmstadt GL, Bhutta ZA, Sultana S, et al. Community-based treatment of serious bacterial infections in newborns and young infants: A randomized controlled trial assessing three antibiotic regimens. Pediatric Infectious Disease Journal. 2012;31:667–72.

8.	Khanal S, Sharma J, GC VS, Dawson P, Houston R, Khadka N, et al. Community health workers can identify and manage possible infections in neonates and young infants: Mini-A model from Nepal. Journal of Health, Population and Nutrition. 2011;29:255–64.

9.	Baqui AH, El-Arifeen S, Darmstadt GL, Ahmed S, Williams EK, Seraji HR, et al. Effect of community-based newborn-care intervention package implemented through two service-delivery strategies in Sylhet district, Bangladesh: a cluster-randomised controlled trial. The Lancet. 2008;371: 1936–44.

10.	Siddiqi S, Kielmann A, Khan M, Ali N, Ghaffar A, Sheikh U, et al. The effectiveness of patient referral in Pakistan. Health Policy and Planning. 2001;16:193–8.

11.	Owais A, Sultana S, Stein AD, Bashir NH, Awaldad R, Zaidi AKM. Why do families of sick newborns accept hospital care a community-based cohort study in Karachi, Pakistan. Journal of Perinatology. 2011;31:586–92.

12.	Duby J, Lassi ZS, Bhutta ZA. Community-based antibiotic delivery for possible serious bacterial infections in neonates in low-and middle-income countries. Cochrane Database of Systematic Reviews. 2019;2019(4) available at https://www.cochranelibrary.com/cdsr/doi/10.1002/14651858.CD007646.pub3/full.

13.	African Neonatal Sepsis Trial (AFRINEST) group, Tshefu A, Lokangaka A, et al. Simplified antibiotic regimens compared with injectable procaine benzylpenicillin plus gentamicin for treatment of neonates and young infants with clinical signs of possible serious bacterial infection when referral is not possible: a randomised, open-label, equivalence trial. Lancet 2015; 385: 1767–76

14.	African Neonatal Sepsis Trial (AFRINEST) group, Tshefu A, Lokangaka A, et al. Oral amoxicillin compared with injectable procaine benzylpenicillin plus gentamicin for treatment of neonates and young infants with fast breathing when referral is not possible: a randomised, open-label, equivalence trial. Lancet 2015; 385: 1758–66

15.	Mir F, Nisar I, Tikmani SS, Baloch B, Shakoor S, Jehan F et al. Simplified antibiotic regimens for treatment of clinical severe infection in the outpatient setting when referral is not possible for young infants in Pakistan (Simplifi ed Antibiotic Therapy Trial [SATT]): a randomised, open-label, equivalence trial. Lancet Glob Health 2017; 5: e177–85

16.	World Health Organization. Guidelines: Managing possible serious bacterial infection in young infants when referral is not feasible. WHO Geneva. 2015.

17.	Ministry of Health and Family Welfare Government of India. Home Based Newborn Care- Operational Guidelines. GoI New Delhi. 2014 available at http://nhsrcindia.org/sites/default/files/Revised%20HBNC%20Operational%20Guidelines%202014%20English.pdf

18.	Ministry of Health and Family Welfare and Ministry of Women and Child Development. Home Based Care for Young Child (HBYC) Operational Guidelines. GoI New Delhi 2018 available at https://www.aspirationaldistricts.in/wp-content/uploads/2019/02/Home-Based-Care-for-Young-Child-Guidelines.pdf

19.	Pathak PK, Singh A, Subramanian S V. Economic inequalities in maternal health care: Prenatal care and skilled birth attendance in India, 1992-2006. PLoS ONE. 2010;5:1992–2006.

20.	Bora JK, Saikia N. Neonatal and under-five mortality rate in Indian districts with reference to Sustainable Development Goal 3: An analysis of the National Family Health Survey of India (NFHS), 2015–2016. PLoS ONE. 2018;13:1–15.

21. Integrated Management of Childhood Illness: management of the sick young infant aged up to 2 months. IMCI chart booklet. Geneva: World Health Organization; 2019. Licence: CC BY-NC-SA 3.0 IGO

22. Degefie Hailegebriel T, Mulligan B, Cousens S, Mathewos B, Wall S, Bekele A, et al. Effect on neonatal mortality of newborn infection management at health posts when referral is not possible: a cluster-randomized trial in rural Ethiopia. Glob Health Sci Pract. 2017; 5(2):202-216. https://doi.org/10.9745/GHSP-D-16-00312
